# Visible-Light/Nickel-Catalyzed Carboxylation of C(sp^2^) Bromides via Formate Activation

**DOI:** 10.1021/acs.joc.3c00895

**Published:** 2023-06-15

**Authors:** Gavin C. Smith, Drason H. Zhang, Wanli Zhang, Abigail H. Soliven, William M. Wuest

**Affiliations:** † Department of Chemistry, Emory University, Atlanta, Georgia 30322, United States; ‡ Department of Chemistry, Purdue University, West Lafayette, Indiana 47907, United States

## Abstract

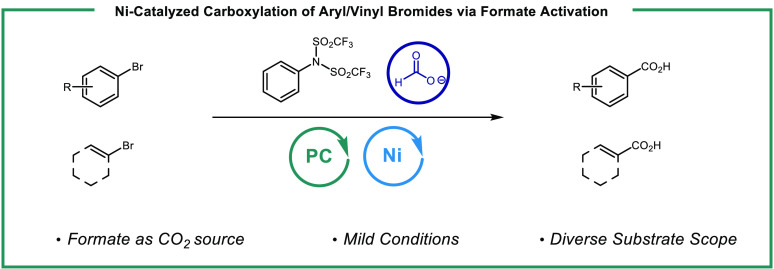

A new visible-light-driven method for the carboxylation of (hetero)aryl/vinyl bromides has been developed using catalytic 4CzIPN, nickel, phenyl triflimide, and sodium formate as a carboxylation agent. Interestingly, we found catalytic phenyl triflimide plays an essential role in promoting the reaction. While many C(sp^2^) carboxylation reactions require harsh reagents or gaseous carbon dioxide, we demonstrate the mild and facile construction of carboxylic acids from readily available starting materials.

Carboxylic acids are one of the most ubiquitous functional groups found across a variety of pharmaceutically and biologically relevant compounds.^1^ Outside of their relevance in bioactive compounds, carboxylic acids are a valuable synthetic linchpin in organic chemistry providing a functional handle for a large range of useful transformations.^2^ Classical methods of constructing carboxylic acids include the addition of organometallic species to CO_2_, oxidation of aldehydes/alcohols, and hydrolysis of esters, amides, and nitriles. While these classical methods remain useful, the utility of catalytic carboxylation reactions with organic halides and CO_2_ has emerged as valuable alternative.^3^ Martin et al. have demonstrated several elegant methods for the carboxylation of aryl (pseudo)halides using either stoichiometric metal reducing agents or dual photocatalytic systems (Figure 1A).^4^ Nickel-catalyzed electrochemical processes have also been demonstrated as valuable alternatives to classical carboxylation reactions (Figure 1B).^5^ We recently disclosed a protocol for accessing the radical anion of carbon dioxide (CO_2_^•–^) through a polarity matched hydrogen atom transfer (HAT) between an electrophilic radical and a formate salt, demonstrating both its nucleophilic and reductive reactivity.^6^ Inspired by the emergence of dual photoredox/nickel catalysis pioneered by Molander, Doyle, and Macmillan,^7^ we sought to expand the reactivity of the CO_2_^•–^ by binding it to a metal center thereby allowing formate salts to replace CO_2_ in C(sp^2^) cross-couplings. Fu et al. recently reported a similar system,^8^ where the use of more highly oxidizing photocatalysts and additives were necessary to access aryl bromide reactivity. In this report, we share our initial insights into this system where we hypothesize phenyl triflimide plays a key role in catalyzing this reaction (Figure 1C).

**Figure 1:**
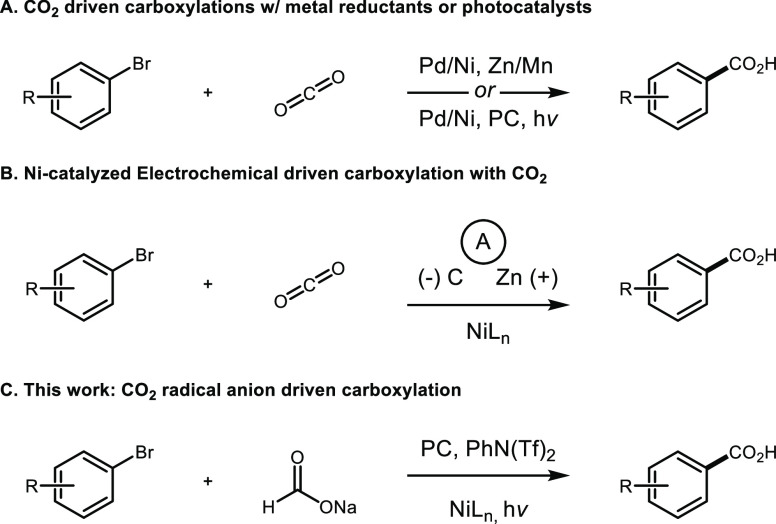
Catalytic strategies for C(sp^2^) carboxylation.

Initial investigations into this transformation with the electron deficient arene, 4-bromobenzonitrile, afforded the carboxylated product in good yield using cyclohexanethiol, sodium formate, 4CzIPN, and NiBr_2_·DTBBPY (see Table S4).

Although many different catalysts may be suitable for this transformation, we opted to use 4CzIPN given its commercial availability and NiBr_2_·DTBBPY given its established efficacy at capturing radicals. These conditions, however, did not translate to electron-rich/neutral arenes using 3-bromoanisole (**A**) and sodium formate (**B**) with several thiol HAT catalysts (Table 1, entries 1–3), indicating there may be unfavorable kinetics between organometallic processes and radical formation. Both 4CzIPN (*E*_1/2_° (PC*/PC^•+^) = −1.2 V vs SCE)^9^ and CO_2_^•–^ (*E*_1/2_° = −2.2 V vs SCE)^6^ are capable of generating the active nickel species (*E*_1/2_^red^[Ni^II^/Ni^0^] = −1.2 V vs SCE),^10^ which may lead to rapid accumulation of Ni(0) followed by catalyst deactivation given the sluggish rate of oxidative addition into electron-rich/neutral halides relative to their electron-deficient counterparts.^11^ Alternatively, given thiol’s proclivity to coordinate metal centers and poison metal catalysts, we postulated that it was likely that thiol was disrupting product formation by interacting with the nickel center.^12^ Although some reports indicate that oxidation of formate to its electrophilic radical renders it a competent HAT catalyst,^8^ control experiments (see Table S3) indicate that formate oxidation is not a viable pathway in this system. Exchanging thiols for the electrophilic HAT catalysts DABCO, HOBt, and NHPI were also not productive in this system (Table 1, entries 4–6). Recognizing that conventional electrophilic HAT catalysts were unlikely to drive this system, we began to explore more creative pathways. In search of a HAT catalyst unlikely to coordinate the metal center, we hypothesized phenyl triflimide, PhN(SO_2_CF_3_)_2_, a reagent commonly used to synthesize enol triflates, may serve as an unorthodox solution to this challenging problem. Oxidation of phenyl triflimide would yield an electrophilic nitrogen centered radical cation capable of HAT; alternatively, reduction of phenyl triflimide would yield a nitrogen centered anion alongside an electrophilic sulfinyl radical.^13^ Gratifyingly, the use of phenyl triflimide in 15 mol % under our standard reaction conditions yielded **3** in near quantitative yield (Table 1, entry 7). Intrigued by this result, we began to further study the role of this unique additive in our reaction system. The standard reduction potential of phenyl triflimide was measured using cyclic voltammetry (see Supporting Information), revealing an irreversible reduction potential (*E*_p/2_ = −0.91 V vs SCE) indicating that single electron transfer (SET) between 4CzIPN (PC*/PC^•+^ = −1.2 V vs SCE) and phenyl triflimide is favorable.

**Table 1:**
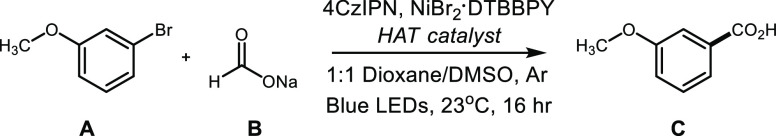
a

entry	HAT catalyst	deviation	yield^b^ (%)
1	cyclohexanethiol	–	0
2	triphenylmethanethiol	–	0
3	triisopropylsilanethiol	–	0
4	DABCO	–	0
5	HOBt	–	0
6	NHPI	–	0
7	PhN(SO_2_CF_3_)_2_	–	98

a Conditions are as follows: Aryl bromide (0.1 mmol), sodium formate (0.15 mmol), 4CzIPN (1 mol %), HAT catalyst (15 mol %), NiBr_2_·dtbbpy (10 mol %), 1:1 DMSO/dioxane (v/v, 0.1 M), blue LEDs, and Ar at 23 °C for 16 h.

b Yields determined via ^1^H NMR using dibromomethane as an internal standard.

Interestingly, replacing phenyl triflimide with methanesulfonyl chloride or toluenesulfonyl chloride resulted in 90% and 76% product yield (Figure 2). Given the parallel reactivity between these three catalytic sulfonylating agents, it is likely they promote analogous reactivity, potentially via SET from the catalyst. Control experiments indicate, however, that phenyl triflimide activates formate for carboxylation (see Supporting Information section V)—a process in which mechanistic studies are currently underway to elucidate the details of this complicated mechanism and the role of phenyl triflimide in this catalytic system.

**Figure 2:**
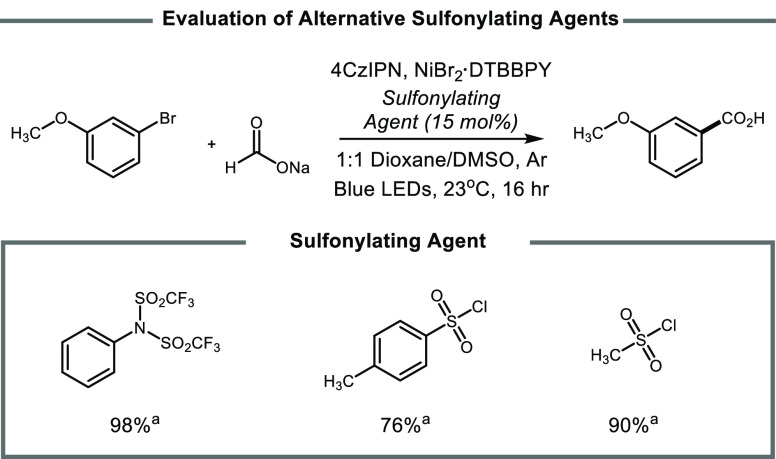
Evaluation of alternative sulfonylating agents in place of phenyl triflimide. ^*a*^Yields determined via ^1^H NMR using dibromomethane as an internal standard.

With our optimized conditions in hand, we sought to demonstrate the utility of this system with a variety of (hetero)aryl/vinyl bromides. We first considered a panel of neutral aryl bromides where bromobenzene, 2-bromotoluene, and 3-bromotoluene all underwent carboxylation in good yield (Table 2, **4**–**6**, 88–91% yield). Interestingly, substrates with ortho substituents were not compatible coupling partners under this system, presumably due to steric effects. We next evaluated a series of electron-rich/electron-poor systems, which all proceeded smoothly to the corresponding carboxylated product (**7**–**10**, 52–98% yield). This system was found to tolerate a variety of functional groups including primary alcohols, ketones, aldehydes, and sulfonamides (**11**–**14**, 51–91% yield). Interestingly, neither aryl ketone **12** nor benzaldehyde **13** showed signs of reduction to the corresponding benzylic alcohols^14^ indicating a preference for CO_2_^•–^ to bind the metal center over SET to the substrate. Furthermore, despite the labile formyl C–H bond^15^ of **13**, we observed no traces of ketone formation from the aldehyde-derived acyl radical.^10^ Halogens were also well tolerated under this system (**15**–**17**, 82–84% yield) where coupling was selective for the C–I bond over the C–Br bond. Finally, we found this system also worked with vinyl bromide **18** (80% yield), although challenges accompanied scaling vinyl bromide substrates (see Supporting Information), alongside a variety of heterocyclic bromides including dioxalane, indole, and indazole further demonstrating the tolerance of this methodology (**19**–**21**, 50–66% yield).

**Table 2:**
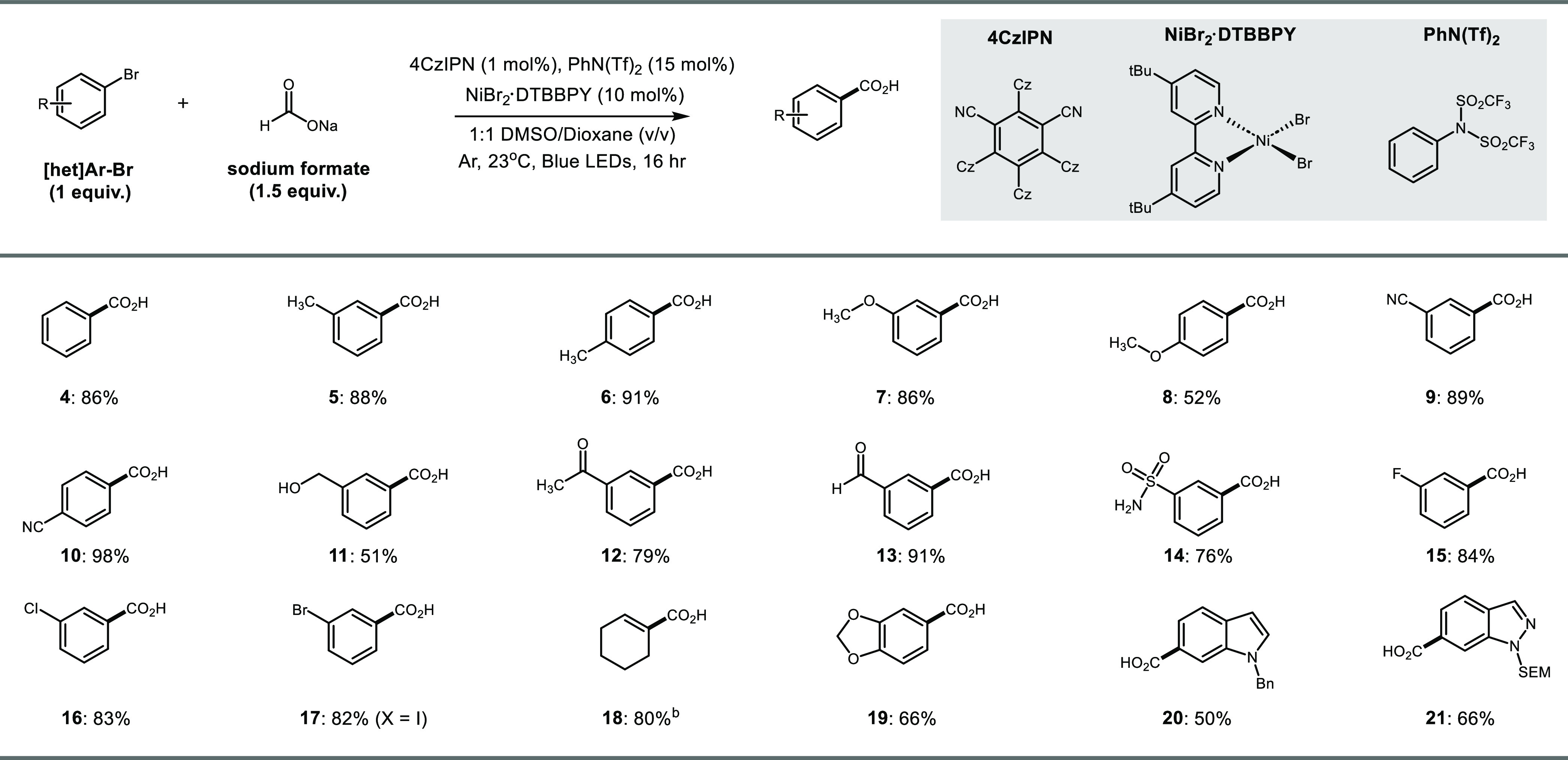
Scope of Ni/Photoredox-Catalyzed Carboxylation of (Hetero)Aryl Bromides and Sodium Formate^a^

a Conditions are as follows: Aryl bromide (1 equiv), sodium formate (1.5 equiv), 4CzIPN (1 mol %), NiBr_2_·dtbbpy (10 mol %), PhN(Tf)_2_ (15 mol %), 1:1 DMSO/dioxane (v/v, 0.1 M), blue LEDs, and Ar at 23 °C for 16 h. Isolated yields are shown unless otherwise stated. Reactions were performed on a 0.5 mmol scale (see Supporting Information).

b Reaction performed on a 0.1 mmol scale; yields determined by ^1^H NMR using CH_2_Br_2_ as an internal standard.

In summary, we have developed a protocol for the carboxylation of C(sp^2^) bromides using abundant formate salts as the CO_2_ source where phenyl triflimide plays a unique role in promoting the reaction. With this information in hand, we demonstrated the carboxylation of a series of electronically distinct (hetero)aryl/vinyl bromides under mild, catalytic conditions. Further mechanistic studies are currently underway to better understand this system and the role of sulfonylating agents in promoting the reaction. Of note, this provides an orthogonal method of installing carboxylate moieties under very mild conditions. We envision that this approach will find broad utility in industry given the benign conditions and cost affective reagents.

## Data Availability

The data underlying this study are available in the published article and its Supporting Information.

## Supporting Information Available

General Information, general procedures, electrochemical measurements, and NMR spectra (PDF)

## References

[ref1] aErtl P.; Altmann E.; Mckenna J. M. The Most Common Functional Groups in Bioactive Molecules and How Their Popularity Has Evolved over Time. J. Med. Chem. 2020, 63, 8408–8418. 10.1021/acs.jmedchem.0c00754. 32663408

[ref2] Gooßen L. J.; Rodríguez N.; Gooßen K. Carboxylic acids as substrates in homogeneous catalysis. Angew. Chem., Int. Ed. 2008, 47, 3100–3120. 10.1002/anie.200704782. 18357604

[ref3] Tortajada A.; Juliá-Hernández F.; Börjesson M.; Moragas T.; Martin R. Transition-Metal-Catalyzed Carboxylation Reactions with Carbon Dioxide. Angew. Chem., Int. Ed. 2018, 130, 16178–16214. 10.1002/ange.201803186. 29722461

[ref4] aCorrea A.; Martín R. Palladium-catalyzed direct carboxylation of aryl bromides with carbon dioxide. J. Am. Chem. Soc. 2009, 131, 15974–15975. 10.1021/ja905264a. 19886688

[ref5] Sun G. Q.; Zhang W.; Liao L. L.; Li L.; Nie Z. H.; Wu J. G.; Zhang Z.; Yu D. G. Nickel-catalyzed electrochemical carboxylation of unactivated aryl and alkyl halides with CO_2_. Nat. Commun. 2021, 12, 1–10. 10.1038/s41467-021-27437-8. 34873172 PMC8648755

[ref6] Hendy C. M.; Smith G. C.; Xu Z.; Lian T.; Jui N. T. Radical Chain Reduction via Carbon Dioxide Radical Anion (CO_2_^•-^). J. Am. Chem. Soc. 2021, 143, 8987–8992. 10.1021/jacs.1c04427. 34102836 PMC8925913

[ref7] aTellis J.; Primer D.; Molander G. Single-electron transmetalation in organoboron cross-coupling by photoredox/nickel dual catalysis. Science 2014, 345, 433–436. 10.1126/science.1253647. 24903560 PMC4406487

[ref8] Fu M. C.; Wang J. X.; Ge W.; Du F. M.; Fu Y. Dual nickel/photoredox catalyzed carboxylation of C(sp^2^)-halides with formate. Org. Chem. Front. 2022, 10, 35–41. 10.1039/D2QO01361D.

[ref9] Speckmeier E.; Fischer T. G.; Zeitler K. A Toolbox Approach to Construct Broadly Applicable Metal-Free Catalysts for Photoredox Chemistry: Deliberate Tuning of Redox Potentials and Importance of Halogens in Donor-Acceptor Cyanoarenes. J. Am. Chem. Soc. 2018, 140, 15353–15365. 10.1021/jacs.8b08933. 30277767

[ref10] Zhang X.; MacMillan D. W. C. Direct Aldehyde C-H Arylation and Alkylation via the Combination of Nickel, Hydrogen Atom Transfer, and Photoredox Catalysis. J. Am. Chem. Soc. 2017, 139, 11353–11356. 10.1021/jacs.7b07078. 28780856 PMC5915308

[ref11] aGisbertz S.; Reischauer S.; Pieber B. Overcoming limitations in dual photoredox/nickel-catalysed C–N cross-couplings due to catalyst deactivation. Nat. Catal. 2020, 3, 611–620. 10.1038/s41929-020-0473-6.

[ref12] aOderinde M. S.; Frenette M.; Robbins D. W.; Aquila B.; Johannes J. W. Photoredox Mediated Nickel Catalyzed Cross-Coupling of Thiols with Aryl and Heteroaryl Iodides via Thiyl Radicals. J. Am. Chem. Soc. 2016, 138, 1760–1763. 10.1021/jacs.5b11244. 26840123

[ref13] de Vleeschouwer F.; van Speybroeck V.; Waroquier M.; Geerlings P.; de Proft F. Electrophilicity and nucleophilicity index for radicals. Org. Lett. 2007, 9, 2721–2724. 10.1021/ol071038k. 17559221

[ref14] Roth H. G.; Romero N. A.; Nicewicz D. A. Experimental and Calculated Electrochemical Potentials of Common Organic Molecules for Applications to Single-Electron Redox Chemistry. Synlett 2016, 27, 714–723. 10.1055/s-0035-1561297.

[ref15] da Silva G.; Bozzelli J. W. Enthalpies of formation, bond dissociation energies, and molecular structures of the n-aldehydes (acetaldehyde, propanal, butanal, pentanal, hexanal, and heptanal) and their radicals. J. Phys. Chem. A 2006, 110, 13058–13067. 10.1021/jp063772b. 17134166

