# Supplementary material for: Visible-Light/Nickel-Catalyzed Carboxylation of C(sp2) Bromides via Formate Activation
Source: J Org Chem. 2023 Jun 15;88(13):9565–8. doi: 10.1021/acs.joc.3c00895 (PMC10337029; doi:10.1021/acs.joc.3c00895)
Supplement: Supplementary file 1 — jo3c00895_si_001.pdf [file jo3c00895_si_001.pdf]

Supporting Information

Visible-Light/Nickel-Catalyzed Carboxylation of C(sp^2) Bromides via Formate Activation

Gavin C. Smith, Drason Zhang, Wanli Zhang, Abigail H. Soliven, William M. Wuest*

[1]Department of Chemistry, Emory University, Atlanta, GA 30322
[2]Department of Chemistry, Purdue University, West Lafayette, Indiana, 47907, United States

Supporting Information

Visible-Light/Nickel-Catalyzed Carboxylation of $C(sp^2)$ Bromides via Formate Activation

Gavin C. Smith, Drason Zhang, Wanli Zhang, Abigail H. Soliven, William M. Wuest*

Department of Chemistry, Emory University, Atlanta, GA 30322
[2]Department of Chemistry, Purdue University, West Lafayette, Indiana, 47907, United States

I. *General Information*……………………………………………S2
a. **General Reagent Information**……………………………………S2
b. **General Analytical Information**…………………………………S2
c. **Abbreviations**……………………………………………………S2
d. **General Photoredox Setup**………………………………………S2
II. *General Procedures*……………………………………………S3
a. **General Procedure A** ……………………………………………S3
III. *Electrochemical Measurements*…………………………………S4
IV. *Optimization Details*……………………………………………S5
V. *Investigations into Phenyl triflimide*……………………………S8
VI. *Preparations of Starting Materials*………………………………S10
VII. *Carboxylation of $C(sp^2)$ Bromides*……………………………S12
VIII. *References*………………………………………………………S19
IX. *NMR spectra*……………………………………………………S21

I. General Information

I-A. General Reagent Information

Reagents were purchased from Sigma-Aldrich, Alfa Aesar, Acros Organics, Combi-Blocks, Oakwood Chemicals, Astatech, and TCI America and used as received, unless stated otherwise. All reactions were set up on the bench top and conducted under nitrogen atmosphere while subject to irradiation from blue LEDs (LEDwholesalers PAR38 Indoor Outdoor 16-Watt LED Flood Light Bulb, Blue; or Hydrofarm® PPB1002 PowerPAR LED Bulb-Blue 15W/E27 (available from Amazon). Flash chromatography was carried out using Siliaflash® P60 silica gel obtained from Silicycle. Thin-layer chromatography (TLC) was performed on 250 μm SiliCycle silica gel F-254 plates. Visualization of the developed chromatogram was performed by fluorescence quenching or staining using $KMnO_4$, p-anisaldehyde, or ninhydrin stains. DMSO was purchased from Fisher Scientific and was distilled over CaH2 and degassed by sonication under vacuum and stored under nitrogen. Photoredox catalyst 4CzIPN was prepared according to literature procedures.[1]

I-B. General Analytical Information.

Unless otherwise noted, all yields refer to chromatographically and spectroscopically (^{1}H NMR) homogenous materials. New compounds were characterized by NMR and HRMS. ^{1}H and ^{13}C NMR spectra were obtained from the Emory University NMR facility and recorded on a Bruker Avance III HD 600 equipped with cryo-probe (600 MHz), Bruker 400 (400 MHz), INOVA 600 (600 MHz), INOVA 500 (500 MHz), INOVA 400 (400 MHz), or VNMR 400 (400 MHz), and are internally referenced to residual protio solvent signals. Data for ^{1}H NMR are reported as follows: chemical shift (ppm), multiplicity (s = singlet, d = doublet, t = triplet, q = quartet, m = multiplet, dd = doublet of doublets, dt = doublet of triplets, ddd= doublet of doublet of doublets, dtd= doublet of triplet of doublets, b = broad, etc.), coupling constant (Hz), integration, and assignment, when applicable. Data for decoupled ^{13}C NMR are reported in terms of chemical shift and multiplicity when applicable. High Resolution mass spectra were obtained from the Emory University Mass Spectral facility using a Thermo Scientific Extractive Plus with an orbitrap mass analyzer.

I-C. Abbreviations.

DMSO = dimethyl sulfoxide
DMF = dimethylformamide
THF = tetrahydrofuran
DCM = dichloromethane
chromatography
TLC = thin layer chromatograph
TEA = triethylamine
EtOAc = ethyl acetate

MeCN = acetonitrile
LCMS = liquid chromatography mass spectrometry
GCMS = gas chromatography mass spectrometry
MeOH = methanol

I-D. General Photoredox Reaction Setup

To run multiple reactions, an appropriately sized 3D printed carousel was used, which exposed the reactions to the blue light evenly (photo 1). While our 3D printed carousel was helpful for screening reactions, it is not necessary in this system—any rack will suffice and our scaled reactions are simply held by a clampl. A 15 W LED array lamp was used as a blue light source (photo 2,3). These lamps were routinely used for up to 12 reactions at a time (photo 2,3). The blue LEDs were positioned approximately 6 inches above the reaction vials to get good light coverage without overheating the reactions (photo 2,3).

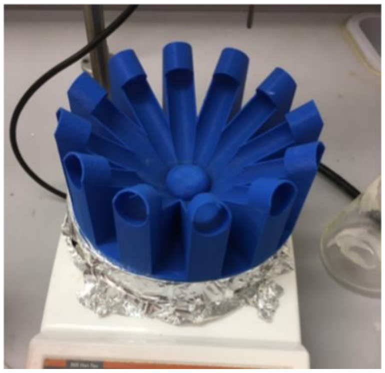

Photo 1

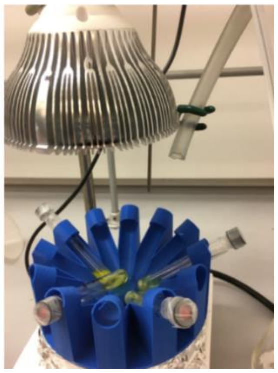

Photo 2

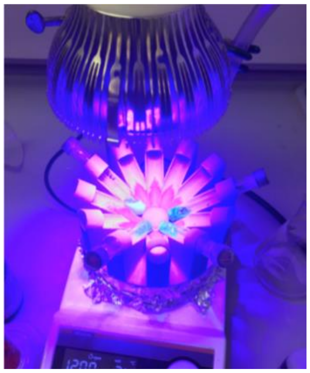

Photo 3

II. General Procedures

General Procedure A

A 20 mL screw-top test tube was charged with 4CzIPN (1 mol%), sodium formate (1.5 equiv), mesna (20 mol%), N-phenyl-bis(trifluoromethanesulfonimide) (15 mol%) and [4,4’-Bis(1,1-dimethyl)-2,2’-bipyridine] nickel (II) bromide (10 mol%)) and substrate (1 equiv., *if solid*). The tube was equipped with a stir bar and was sealed with a PTFE/silicon septum. The atmosphere was exchanged by applying vacuum and backfilling with argon (this process was conducted a total of three times). Under argon atmosphere, separately the indicated degassed solvent (0.1 M) was added via syringe followed by the substrate (*if liquid*, 1.0 equiv). The resulting mixture was stirred at 1400 RPM for 16 h under irradiation by blue LEDs. 1M HCl was added and then the reaction mixture was extracted with ethyl acetate (3 x). The organic layer was dried over $MgSO_4$ and concentrated. The residue was purified on silica using the indicated solvent mixture as eluent to afford the title compound.

III. Electrochemical Measurements

Electrochemical potentials were obtained with a standard set of conditions according to literature procedure.[1] Cyclic voltammograms (CVs) were collected with a VersaSTAT 4Potentiostat. Samples were prepared with 0.1 mmol of substrate in 10 mL of 0.1 M tetra-n-butylammonium hexafluorophosphate in dry, degassed acetonitrile. Measurements employed a glassy carbon working electrode, platinum wire counter electrode, 3M NaCl silver-silver chloride reference electrode, and a scan rate of 100 mV/s. Reductions were measured by scanning potentials in the negative direction and oxidations in the positive direction; the glassy carbon electrode was polished between each scan. Data was analyzed using Microsoft Excel by subtracting a background current prior to identifying the maximum current (Cp) and determining the potential (Ep/2) at half this value (Cp/2). The obtained value was referenced to Ag|AgCl and converted to SCE by subtracting 0.035 V.

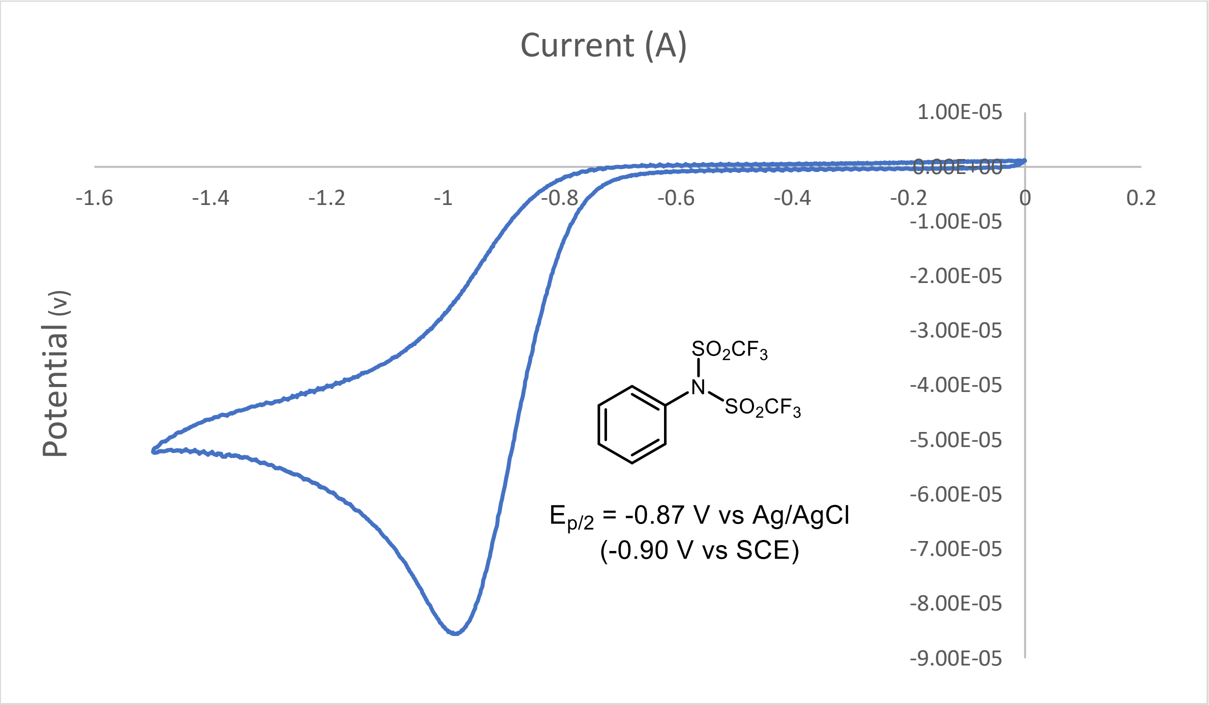

IV. Optimization Details

Optimization Procedure

A 20 mL screw-top test tube was charged with 4CzIPN (1 mol%), sodium formate (1.5 equiv), mesna (20 mol%), N-phenyl-bis(trifluoromethanesulfonimide) (15 mol%) and [4,4'-Bis(1,1-dimethyl)-2,2'-bipyridine] nickel (II) bromide (10 mol%)) and substrate (1 equiv., *if solid*). The tube was equipped with a stir bar and was sealed with a PTFE/silicon septum. The atmosphere was exchanged by applying vacuum and backfilling with argon (this process was conducted a total of three times). Under argon atmosphere, separately the indicated degassed solvent (0.1 M) was added via syringe followed by the substrate (*if liquid*, 1.0 equiv). The resulting mixture was stirred at 1400 RPM for 16 h under irradiation by blue LEDs. 1M HCl was added and then the reaction mixture was extracted with ethyl acetate (3 x). The organic layer was dried over $MgSO_4$ and concentrated. Deutro-chloroform with an internal standard of dibromomethane (7 µL, 0.1 mmol) was added. The sample was analyzed by ^1H NMR (d = 5 s), and the integral values were used to calculate the data given in Table S1- S3.

HAT Catalysts:

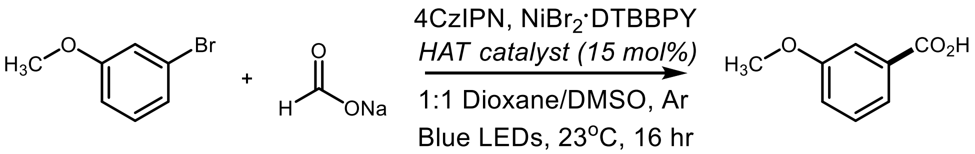

Entry	HAT Catalyst	Deviation	Yield[a]
1	Cyclohexanethiol	-	0%
2	Triphenylmethanethiol	-	0%
3	Triisopropylsilanethiol	-	0%
4	DABCO	-	0%
5	HOBt	-	0%
6	$PhN(SO_2CF_3)_2$	-	98%
7	Methanesulfonyl Chloride	-	90%
8	p-toluenesulfonyl chloride	-	76%
9	Sodium p-toluenesulfinate	-	0%

Table S1

Solvent Screen

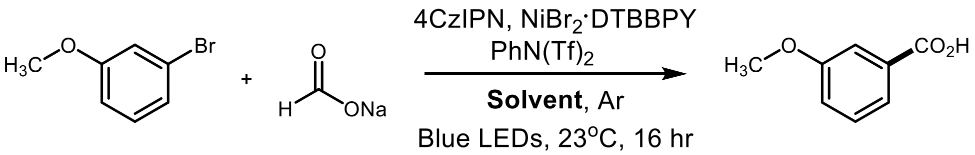

Entry	Solvent	Yield
1	1:1 Dioxane/DMSO	98%
2	Dioxane	0%
3	DMSO	85%
4	DMF	34%

Table S-2

Control Experiments:

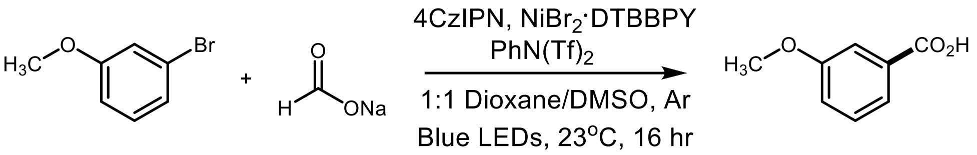

Entry	Deviation	Yield
1	No 4CzIPN	0%
2	No $PhN(Tf)_2$	0%
3	No $NaHCO_2$	0%
4	No light	0%
5	No air-free precautions	60%

Table S3.

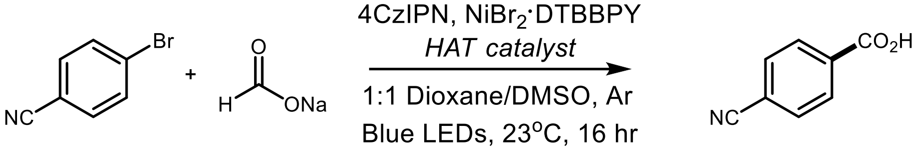

1 **2** **3**

Entry	HAT Catalyst	Deviation	Yield[a]
1	Cyclohexanethiol	-	75%
2	Mesna	-	30%
3	Thiophenol	-	73%
4	Tert-butylthiol	-	51%
5	Thioacetic Acid	-	57%

Table S-4

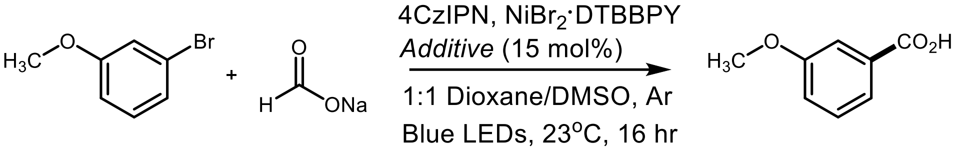

1 **2** **3**

Entry	HAT Catalyst	Deviation	Yield[a]
1	Trifluoro-*N*-phenylmethanesulfonamide	-	0%
2	Sodium trifluoromethanesulfonate	-	0%
3	Sodium p-toluenesulfinate	-	0%

Table S-5

V. Investigations into the Role of Phenyl Triflimide

Samples were prepared using 0.015 mmol of phenyl triflimide (**A**) alongside a combination of 0.015 mmol phenyl triflimide and 0.15 mmol sodium formate (**B**) using 1-bromo-3-fluorobenzene as an internal standard in DMSO-d_6.

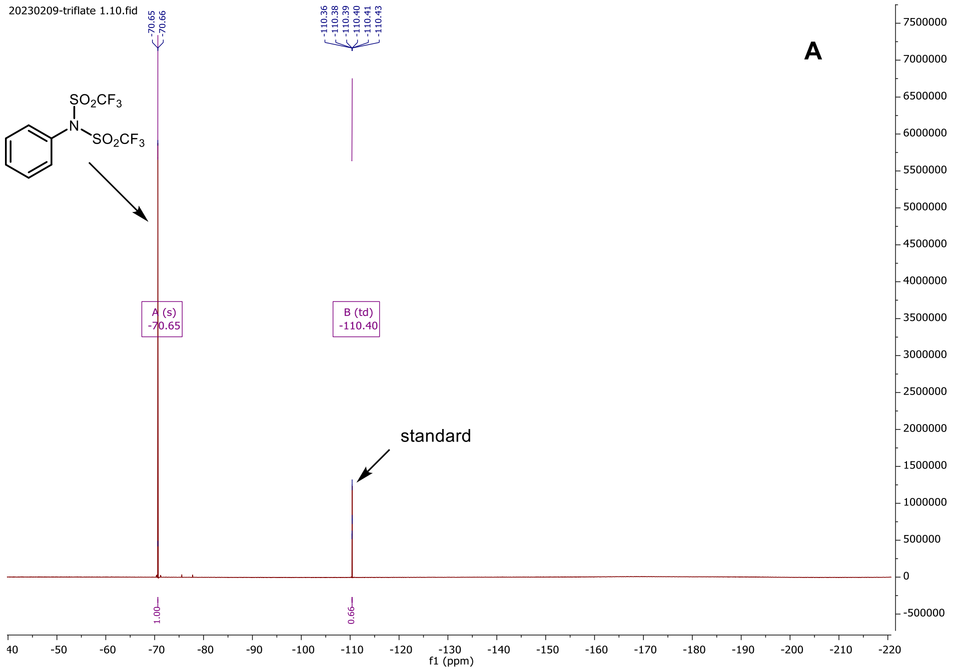

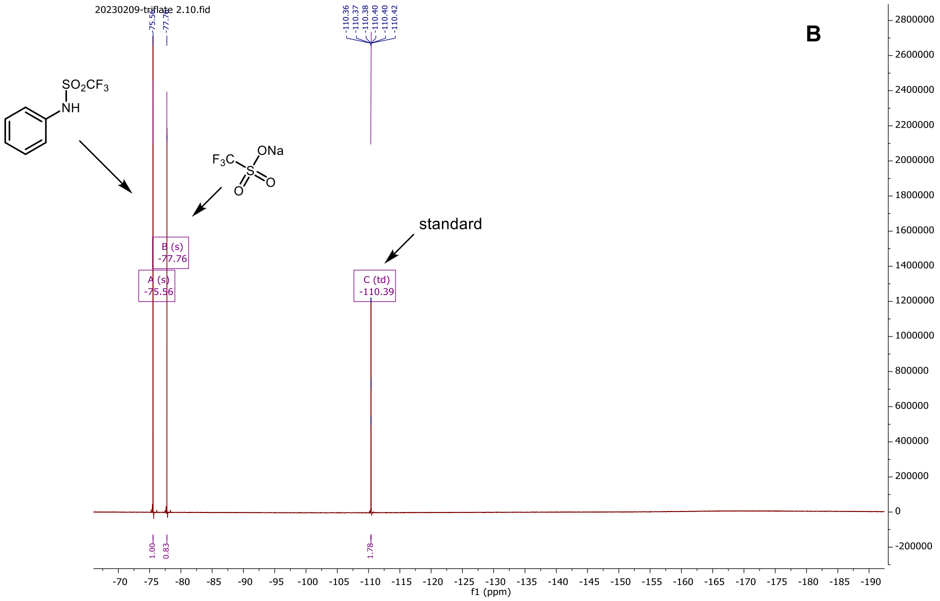

Spectra B shows trifluoro-N-phenylmethanesulfonamide at -75.56 ppm alongside sodium trifluoromethanesulfonate at -77.76 ppm, these chemical shifts are consistent with the literature spectra.[21] The presence of trifluoro-N-phenylmethanesulfonamide and sodium trifluoromethanesulfonate when sodium formate and phenyl triflimide are in solution together suggests the following reaction pathway.

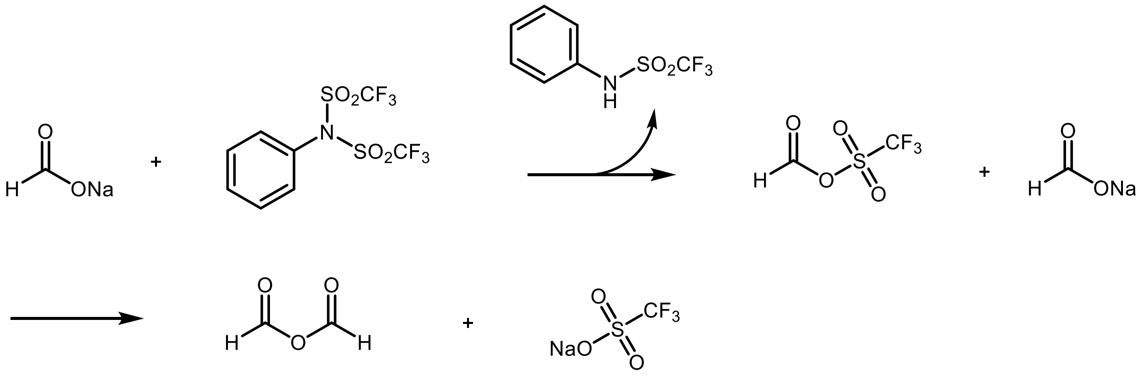

In this reaction pathway, trifluoro-N-phenylmethanesulfonamide and sodium trifluoromethanesulfonate are generated—when tested independently as additives (**Table S-4**) no product formation is observed indicating that *in situ* generated formic anhydride may be playing a role by masking formate as the anhydride. A potential role for formic anhydride is shown below.

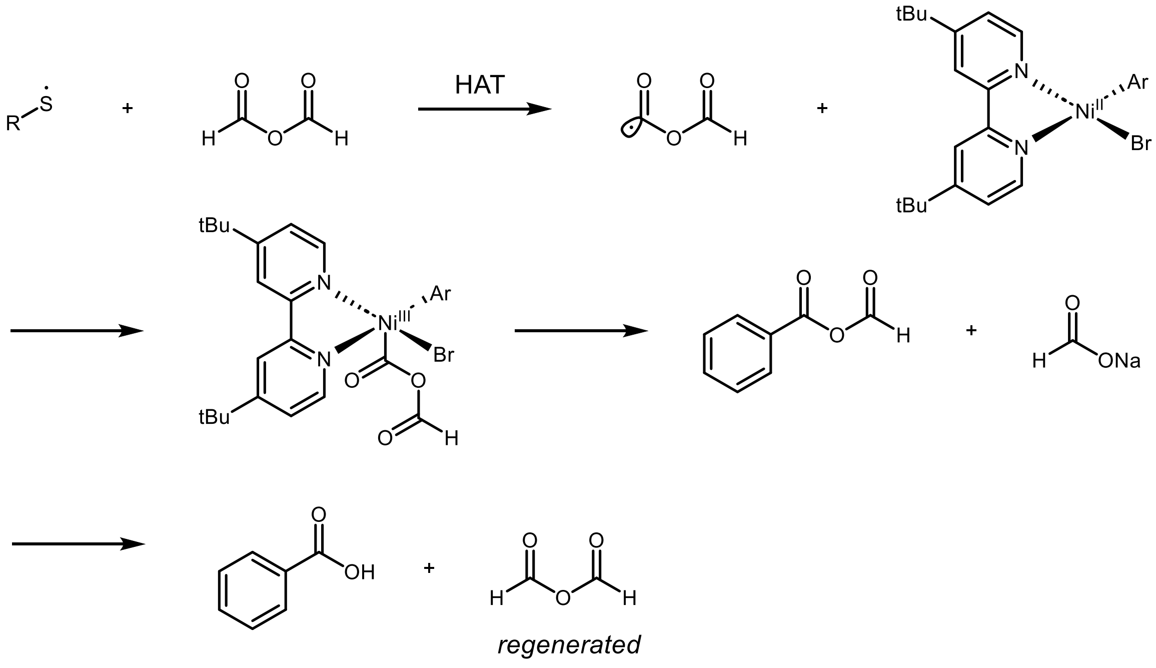

In this mechanistic scenario, DMSO may serve as a source of electrophilic HAT catalysts in the form of thiol. Our previous work has demonstrated that DMSO decomposes to various thiol/sulfur based impurities that catalyze radical formation[22]. HAT from formic anhydride would generate the acyl radical that may bind the metal center. Upon reductive elimination the mixed anhydride is formed which may be hydrolyzed by formate to release the carboxylate while reforming formic anhydride. Given phenyl triflimide is catalytic in this reaction (15 mol%), formic anhydride must be regenerated. Masking formate as the anhydride may prevent undesired reduction chemistry from the carbon dioxide radical anion to substrates or metal centers making this reaction more efficacious. Given the complexities of this system and how phenyl triflimide

and its degradation products fit into the catalytic cycles, further mechanistic studies are required to elucidate the details of this mechanism.

VI. Preparation of Starting Materials

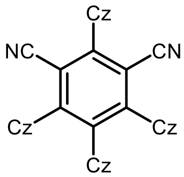

2,4,5,6-Tetrakis(carbazole-9-yl)-4,6-dicyanobenzene (4CzIPN) (S1): To a flame dried round bottom flask was added carbazole (1.67 g, 10.0 mmol) in anhydrous THF (40 mL). Sodium hydride (60% in oil, 0.60 g, 15.0 mmol) was carefully added portion wise to the solution. After 30 minutes, tetrafluoroisopthalonitrile (0.40 g, 2.00 mmol) was added and allow to stir at room temperature for 16 h. Water (2 mL) was then added carefully and the reaction mixture was then concentrated *in vacuo*. The resulting solid was then washed with water and ethanol then recrystallized from hexanes/CH_2Cl_2 to yield the product was a vibrant yellow solid (1.4 g, 89% yield). The physical and spectral properties were consistent with reported values.[3]
^{1}H NMR (400 MHz, $CDCl_3$) δ 8.21 (d, J = 7.8 Hz, 2H), 7.75-7.65 (m, 8H), 7.52-7.45 (m, 2H), 7.33 (d, J = 7.5 Hz, 2H), 7.24-7.19 (m, 4H), 7.12-7.03 (m, 8H), 6.86-6.79 (m, 4H) 6.69-6.61 (m, 2H).

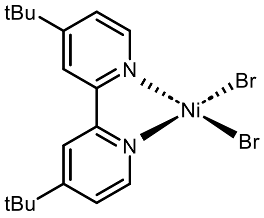

[4,4'-Bis(1,1-dimethyl)-2,2'-bipyridine] nickel (II) bromide (S2): Following a previously reported synthesis, a flame-dried round bottom flask was charged with $NiBr_2$(glyme) (0.616 g, 2.0 mmol, 1 equiv.) and 4,4'-di-*tert*-butyl-2,2'-bipyridyl (0.536 g, 2.0 mmol, 1 equiv.) before being placed under argon. Anhydrous THF (60 mL) was added and the reaction mixture was stirred for 20 h. The resulting green solid was filtered off and washed with diethyl ether and dried under vacuum to afford the title compound as a green powder that was used without further purification (0.89g, 91% yield).[20]
1H NMR (400 MHz, DMSO-d_6) δ 6.04 (1.53, 9H). The paramagnetic product shows only a broad peak at 1.53 ppm.[20]

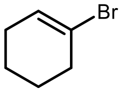

1-bromocylcohex-1-ene (S3): To a flame-dried round bottom flask was added triphenyl phosphite (6.8 g, 22.0 mmol, 1.1 equiv.). The atmosphere was exchanged three times with argon and equipped with an argon balloon. Anhydrous CH_2Cl_2 was added (60 mL) and the reaction

was cooled to -78°C. Bromine (1.2 mL, 24.0 mmol, 1.2 equiv.) was added slowly and allowed to stir for five minutes before the slow addition of triethylamine (3.6 mL, 26.0 mmol, 1.3 equiv.). The reaction mixture was stirred for another five minutes before the addition of cyclohexanone (2.1 mL, 20.0 mmol, 1 equiv.). The reaction mixture was warmed to room temperature overnight then refluxed in an oil bath for two hours. The crude reaction mixture was then concentrated and purified by silica chromatography (100% Hexanes as the eluent) to afford the title compound as a pale yellow oil (1.83 g, 56% yield). The physical and spectral properties match the reported values.[4]

^{1}H NMR (400 MHz, CDCl$_3$) δ 6.04 (m, 1H), 2.42 (m, 2H), 2.08-2.07 (m, 2H)), 1.77-1.71 (m, 2H), 1.64-1.58 (m, 2H).

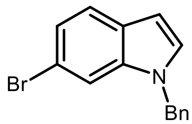

1-benzyl-6-bromo-1*H*-indole (S4): To a flame-dried round bottom flask was added 6-bromoindole (0.98 g, 5.0 mmol, 1 equiv.) followed by anhydrous DMF (5 mL). The reaction mixture was cooled to 0°C and sodium hydride (0.24 g, 6.0 mmol, 1.2 equiv.) portion wise. Stir for thirty minutes at 0oC then add benzyl bromide (0.9 mL, 7.5 mmol, 1.5 equiv., dissolved in 2.5 mL DMF) slowly. The reaction mixture was warmed to room temperature and stirred overnight. After cooling back down to 0°C, the reaction was quenched with water (5 mL), extracted three times with ethyl acetate, dried over MgSO4 and concentrated *in vacuo*. The crude reaction mixture was then purified by silica chromatography (2.5 % EtOAc/Hexanes as the eluent) to afford the title compound as an off white solid (1.08 g, 76% yield). The physical and spectral properties match the reported values.[5]

^{1}H NMR (400 MHz, CDCl$_3$) δ 7.50 (d, J = 8.4 Hz, 1H), 7.45-7.41 (m, 1H), 7.36-7.27 (m, 3H), 7.21 (dd, J = 8.4 Hz, 1.7 Hz, 1H), 7.12-7.03 (m, 3H), 6.52 (dd, J = 3.1, 1.0 Hz, 1H), 5.28 (s, 2H).

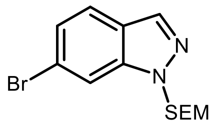

6-bromo-1-((2-(trimethylsilyl)ethoxy)methyl)-1*H*-indazole (S5): To a round bottom flask was added 6-bromoindazole (0.493 g, 2.5 mmol, 1 equiv.) and CH_2Cl_2 (10 mL). The reaction mixture was cooled to 0°C and potassium hydroxide (0.168 g, 3.0 mmol, 3 equiv., dissolved in 0.6 mL H_2O) was added followed by tetrabutylammonium bromide (0.080 g, 0.25 mmol, 0.1 equiv.). SEM-chloride (0.49 mL, 2.75 mmol, 1.1 equiv.) was then added dropwise and stirred at 0°C for 1 hour. The reaction mixture was allowed to warm to room temperature, stirred overnight, quenched with H_20 (10 mL) and extracted three times with CH_2Cl_2. The crude reaction mixture was dried over MgSO4, concentrated *in vacuo*, and purified via silica chromatography (5-10% EtOAc/Hexanes as the eluent) to afford the title compound as a light brown oil (0.412 g, 50% yield).

^{1}H NMR (400 MHz, CDCl$_3$) δ 7.97 (d, J = 1.0 Hz, 1H), 7.79-7.77 (m, 1H), 7.60 (dd, J = 8.6 Hz, 0.7 Hz, 1H), 7.30 (dd, J = 8.5 Hz, 1.6 Hz, 1H), 5.70 (s, 2H), 3.56-3.50 (m, 2H), 0.91-0.85 (m, 2H), -0.06 (s, 9H).

^{13}C{^{1}H} NMR (100 MHz, CDCl$_3$) δ 140.6, 134.2, 125.1 123.7, 122.3, 121.3, 112.9, 77.9, 66.6

17.8, -1.1, -1.3, -1.6.
HRMS (APCI) *m/z*: [M+] calcd. for $C_{12}H_{20}ON_2Si$, 327.0523, found 327.0520.
R_f: 0.47 (5% EtOAc/Hexanes)

VII. Preparation of Products from Substrate Table

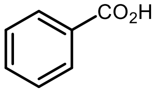

Benzoic acid (4): Prepared according to the general procedure using bromobenzene (0.5 mmol, 53 µl, 1 equiv.), sodium formate (0.75 mmol, 0.051 g, 1.5 equiv.), 4CzIPN (.005 mmol, 0.0039 g, 1 mol%), N-phenyl-bis(trifluoromethanesulfonimide) (0.075 mmol, 0.027 g, 15 mol%), and [4,4'-Bis(1,1-dimethyl)-2,2'-bipyridine] nickel (II) bromide (0.05 mmol, 0.024 g, 10 mol%), in 1:1 DMSO/dioxane (5 mL). After 16 hours the reaction was quenched with 1M HCl (10 mL), extracted three times with ethyl acetate, dried over $MgSO_4$ and concentrated *in vacuo*. The crude reaction mixture was purified by silica chromatography (10% EtOAc/hexanes + 1% AcOH as the eluent) to afford the title compound as a pale yellow solid (0.065 g, 86% yield). The physical and spectral properties were consistent with the reported values.[6]
^{1}H NMR (400 MHz, DMSO-d$_6$) δ 12.94 (s, 1H), 7.95 (d, J = 7.3 Hz, 2H), 7.62 (t, J = 7.3 Hz , 2H), 7.50 (t, J = 7.7 Hz, 1H).

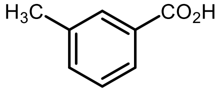

3-methylbenzoic acid (5): Prepared according to the general procedure using 3-bromotoluene (0.5 mmol, 61 µl, 1 equiv.), sodium formate (0.75 mmol, 0.051 g, 1.5 equiv.), 4CzIPN (.005 mmol, 0.0039 g, 1 mol%), N-phenyl-bis(trifluoromethanesulfonimide) (0.075 mmol, 0.027 g, 15 mol%), and [4,4'-Bis(1,1-dimethyl)-2,2'-bipyridine] nickel (II) bromide (0.05 mmol, 0.024 g, 10 mol%), in 1:1 DMSO/dioxane (5 mL). After 16 hours the reaction was quenched with 1M HCl (10 mL), extracted three times with ethyl acetate, dried over $MgSO_4$ and concentrated *in vacuo*. The crude reaction mixture was purified by silica chromatography purified by silica chromatography (20% EtOAc/hexanes + 1% AcOH as the eluent) to afford the title compound as a whitea solid (0.060 g, 88% yield). The physical and spectral properties were consistent with the reported values.[7]
^{1}H NMR (600 MHz, CDCl$_3$) δ 7.95-7.90 (m, 2H), 7.43 (d, J = 7.6z Hz, 2H), 7.38 (t, J = 7.4 Hz , 1fH), 2.43 (s, 3H).

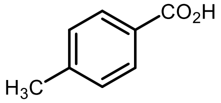

4-methylbenzoic acid (6): Prepared according to the general procedure using 4-bromotoluene (0.5 mmol, 61 µl, 1 equiv.), sodium formate (0.75 mmol, 0.051 g, 1.5 equiv.), 4CzIPN (.005 mmol, 0.0039 g, 1 mol%), N-phenyl-bis(trifluoromethanesulfonimide) (0.075 mmol, 0.027 g, 15 mol%), and [4,4'-Bis(1,1-dimethyl)-2,2'-bipyridine] nickel (II) bromide (0.05 mmol, 0.024 g, 10

mol%), in 1:1 DMSO/dioxane (5 mL). After 16 hours the reaction was quenched with 1M HCl (10 mL), extracted three times with ethyl acetate, dried over $MgSO_4$ and concentrated *in vacuo*. The crude reaction mixture was purified by silica chromatography purified by silica chromatography (20% EtOAc/hexanes + 1% AcOH as the eluent) to afford the title compound as a white solid (0.062 g, 91% yield). The physical and spectral properties were consistent with the reported values.[8]
^{1}H NMR (600 MHz, $CDCl_3$) δ 7.95-7.90 (m, 2H), 7.43 (d, J = 7.6 Hz, 2H), 7.38 (t, J = 7.4 Hz , 1fH), 2.43 (s, 3H).

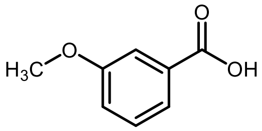

3-methoxybenzoic acid (7): Prepared according to the general procedure using 3-bromoanisole (0.5 mmol, 63 µl, 1 equiv.), sodium formate (0.75 mmol, 0.051 g, 1.5 equiv.), 4CzIPN (.005 mmol, 0.0039 g, 1 mol%), N-phenyl-bis(trifluoromethanesulfonimide) (0.075 mmol, 0.027 g, 15 mol%), and [4,4'-Bis(1,1-dimethyl)-2,2'-bipyridine] nickel (II) bromide (0.05 mmol, 0.024 g, 10 mol%), in 1:1 DMSO/dioxane (5 mL). After 16 hours the reaction was quenched with 1M HCl (10 mL), extracted three times with ethyl acetate, dried over $MgSO_4$ and concentrated *in vacuo*. The crude reaction mixture was purified by silica chromatography purified by silica chromatography (20-30% EtOAc/hexanes + 1% AcOH as the eluent) to afford the title compound as a pale yellow solid (0.065 g, 86% yield). The physical and spectral properties were consistent with the reported values.[9]
^{1}H NMR (400 MHz, DMSO-d_6) δ 13.00 (s, 1H), 7.53 (dt, J = 7.6, 1.3 Hz, 1H), 7.44-7.38 (m, 2H), 7.18 (ddd, J = 8.20, 2.7, 1.0 Hz, 1H), 3.80 (s, 3H).

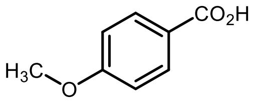

4-methoxybenzoic acid (8): Prepared according to the general procedure using 4-bromoanisole (0.5 mmol, 63 µl, 1 equiv.), sodium formate (0.75 mmol, 0.051 g, 1.5 equiv.), 4CzIPN (.005 mmol, 0.0039 g, 1 mol%), N-phenyl-bis(trifluoromethanesulfonimide) (0.075 mmol, 0.027 g, 15 mol%), and [4,4'-Bis(1,1-dimethyl)-2,2'-bipyridine] nickel (II) bromide (0.05 mmol, 0.024 g, 10 mol%), in 1:1 DMSO/dioxane (5 mL). After 16 hours the reaction was quenched with 1M HCl (10 mL), extracted three times with ethyl acetate, dried over $MgSO_4$ and concentrated *in vacuo*. The crude reaction mixture was purified by silica chromatography purified by silica chromatography (20-30% EtOAc/hexanes + 1% AcOH as the eluent) to afford the title compound as a pale yellow solid (0.039 g, 52% yield). The physical and spectral properties were consistent with the reported values.[9]
^{1}H NMR (400 MHz, DMSO-d_6) δ 13.00 (s, 1H), 7.89 (d, J = 8.9, 2H), 7.02 (d, J = 8.8 Hz, 2H), 3.82 (s, 3H).

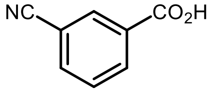

3-cyanobenzoic acid (9): Prepared according to the general procedure using 3-bromobenzonitrile (0.5 mmol, 0.091g, 1 equiv.), sodium formate (0.75 mmol, 0.051 g, 1.5 equiv.), 4CzIPN (.005 mmol, 0.0039 g, 1 mol%), N-phenyl-bis(trifluoromethanesulfonimide) (0.075 mmol, 0.027 g, 15 mol%), and [4,4'-Bis(1,1-dimethyl)-2,2'-bipyridine] nickel (II) bromide (0.05 mmol, 0.024 g, 10 mol%), in 1:1 DMSO/dioxane (5 mL). After 16 hours the reaction was quenched with 1M HCl (10 mL), extracted three times with ethyl acetate, dried over $MgSO_4$ and concentrated *in vacuo*. The crude reaction mixture was purified by silica chromatography purified by silica chromatography (20-30% EtOAc/hexanes + 1% AcOH as the eluent) to afford the title compound as a white solid (0.062 g, 89% yield). The physical and spectral properties were consistent with the reported values.[10]
^{1}H NMR (400 MHz, DMSO-d_6) δ 13.00 (s, 1H), 8.30-8.26 (m 1H), 8.25-8.20 (dt, J = 7.9, 1.4 Hz, 1H), 8.13-8.07 (dt, J = 7.7, 1.3, 1H), 7.73 (t, J = 7.8 Hz, 1H).

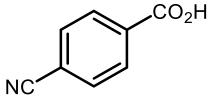

4-cyanobenzoic acid (10): Prepared according to the general procedure using 4-bromobenzonitrile (0.5 mmol, 0.091g, 1 equiv.), sodium formate (0.75 mmol, 0.051 g, 1.5 equiv.), 4CzIPN (.005 mmol, 0.0039 g, 1 mol%), N-phenyl-bis(trifluoromethanesulfonimide) (0.075 mmol, 0.027 g, 15 mol%), and [4,4'-Bis(1,1-dimethyl)-2,2'-bipyridine] nickel (II) bromide (0.05 mmol, 0.024 g, 10 mol%), in 1:1 DMSO/dioxane (5 mL). After 16 hours the reaction was quenched with 1M HCl (10 mL), extracted three times with ethyl acetate, dried over $MgSO_4$ and concentrated *in vacuo*. The crude reaction mixture was purified by silica chromatography purified by silica chromatography (20-30% EtOAc/hexanes + 1% AcOH as the eluent) to afford the title compound as a pale yellow solid (0.073 g, 98% yield). The physical and spectral properties were consistent with the reported values.[11]
^{1}H NMR (400 MHz, $CDCl_3$) δ 8.22 (d, J = 8.2 Hz, 2H), 7.80 (d, J = 8.2 Hz, 2H).)

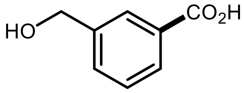

3-(hydroxymethyl)benzoic acid (**11**): Prepared according to the general procedure using 3-bromobenzyl alcohol (0.5 mmol, 60 µl, 1 equiv.), sodium formate (0.75 mmol, 0.051 g, 1.5 equiv.), 4CzIPN (.005 mmol, 0.0039 g, 1 mol%), N-phenyl-bis(trifluoromethanesulfonimide) (0.075 mmol, 0.027 g, 15 mol%), and [4,4'-Bis(1,1-dimethyl)-2,2'-bipyridine] nickel (II) bromide (0.05 mmol, 0.024 g, 10 mol%), in 1:1 DMSO/dioxane (5 mL). After 16 hours the reaction was quenched with 1M HCl (10 mL), extracted three times with ethyl acetate, dried over $MgSO_4$ and concentrated *in vacuo*. The crude reaction mixture was purified by silica chromatography purified by silica chromatography (20-50% EtOAc/hexanes + 1% AcOH as the eluent) to afford the title compound as a white solid (0.039 g, 51% yield).
^{1}H NMR (400 MHz, DMSO-d_6) δ 7.92 (s, 1H), 7.81 (d, J = 7.7 Hz, 1H), 7.54 (d, J = 7.7 Hz, 1H) 7.44 (t, J = 7.6 Hz, 1H) 5.32 (bs, 1H), 4.55 (s, 2H).
^{13}C {^{1}H} NMR (100 MHz, $CDCl_3$) δ 167.4, 143.1 130.8, 130.7, 128.3, 127.6, 127.2, 62.4.
HRMS (APCI) *m/z*: [M+H] calcd. for $C_8H_9O_3$, 153.0546, found 153.0546.
R_f: 0.36 (40% EtOAc/Hexanes + 1% AcOH).

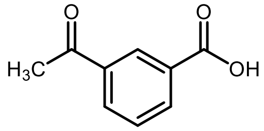

3-acetylbenzoic acid (12): Prepared according to the general procedure using 3-bromoacetophenone (0.5 mmol, 66 µl, 1 equiv.), sodium formate (0.75 mmol, 0.051 g, 1.5 equiv.), 4CzIPN (.005 mmol, 0.0039 g, 1 mol%), N-phenyl-bis(trifluoromethanesulfonimide) (0.075 mmol, 0.027 g, 15 mol%), and [4,4'-Bis(1,1-dimethyl)-2,2'-bipyridine] nickel (II) bromide (0.05 mmol, 0.024 g, 10 mol%), in 1:1 DMSO/dioxane (5 mL). After 16 hours the reaction was quenched with 1M HCl (10 mL), extracted three times with ethyl acetate, dried over $MgSO_4$ and concentrated *in vacuo*. The crude reaction mixture was purified by silica chromatography purified by silica chromatography (20-30% EtOAc/hexanes + 1% AcOH as the eluent) to afford the title compound as a white solid (0.065 g, 79% yield). The physical and spectral properties were consistent with the reported values.[12]
^{1}H NMR (400 MHz, $CDCl_3$) δ 8.68 (s, 1H), 8.32 (d, J = 7.7 Hz, 1H), 8.23 (d, J = 7.9 Hz, 1H), 7.62 (t, J = 7.8 Hz, 1H), 2.68 (s, 3H).

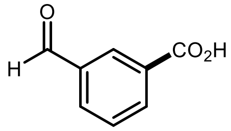

3-formylbenzoic acid (13): Prepared according to the general procedure using 3-bromobenzaldehyde (0.5 mmol, 0.093 g, 1 equiv.), sodium formate (0.75 mmol, 0.051 g, 1.5 equiv.), 4CzIPN (.005 mmol, 0.0039 g, 1 mol%), N-phenyl-bis(trifluoromethanesulfonimide) (0.075 mmol, 0.027 g, 15 mol%), and [4,4'-Bis(1,1-dimethyl)-2,2'-bipyridine] nickel (II) bromide (0.05 mmol, 0.024 g, 10 mol%), in 1:1 DMSO/dioxane (5 mL). After 16 hours the reaction was quenched with 1M HCl (10 mL), extracted three times with ethyl acetate, dried over $MgSO_4$ and concentrated *in vacuo*. The crude reaction mixture was purified by silica chromatography purified by silica chromatography (20% EtOAc/hexanes + 1% AcOH as the eluent) to afford the title compound as a pale yellow solid (0.068 g, 91% yield). The physical and spectral properties were consistent with the reported values.[13]
^{1}H NMR (400 MHz, DMSO-d_6) δ 13.37 (s, 1H), 10.09 (s, 1H), 8.44 (s, 1H), 8.24 (d, J = 7.6 Hz, 1H), 8.14 (d, J = 7.7 Hz, 1H), 7.74 (t, J = 7.6 Hz, 1H).

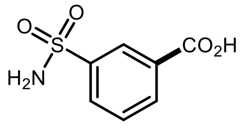

3-sulfamoylbenzoic acid (14): Prepared according to the general procedure using 3-bromobenzenesulfonamide (0.5 mmol, 0.118 g, 1 equiv.), sodium formate (0.75 mmol, 0.051 g, 1.5 equiv.), 4CzIPN (.005 mmol, 0.0039 g, 1 mol%), N-phenyl-bis(trifluoromethanesulfonimide) (0.075 mmol, 0.027 g, 15 mol%), and [4,4'-Bis(1,1-dimethyl)-2,2'-bipyridine] nickel (II)

bromide (0.05 mmol, 0.024 g, 10 mol%), in 1:1 DMSO/dioxane (5 mL). After 16 hours the reaction was quenched with 1M HCl (10 mL), extracted three times with ethyl acetate, dried over $MgSO_4$ and concentrated *in vacuo*. The crude reaction mixture was purified by silica chromatography purified by silica chromatography (0-50% EtOAc/hexanes + 1% AcOH followed by 0-10% MeOH/CH_2Cl_2 as the eluent) to afford the title compound as a pale yellow solid (0.077 g, 76% yield). The physical and spectral properties were consistent with the reported values.[14]
^{1}H NMR (400 MHz, DMSO-d_6) δ 13.42 (bs, 1H), 8.39 (s, 1H), 8.14 (d, J = 7.8 Hz, 1H), 8.05 (d, J = 8.1 Hz, 1H), 7.71 (t, J = 7.7 Hz, 1H), 7.50 (s, 2H).

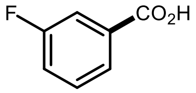

3-fluorobenzoic acid (15): Prepared according to the general procedure using 1-bromo-3-fluorobenzene (0.5 mmol, 56 µl, 1 equiv.), sodium formate (0.75 mmol, 0.051 g, 1.5 equiv.), 4CzIPN (.005 mmol, 0.0039 g, 1 mol%), N-phenyl-bis(trifluoromethanesulfonimide) (0.075 mmol, 0.027 g, 15 mol%), and [4,4'-Bis(1,1-dimethyl)-2,2'-bipyridine] nickel (II) bromide (0.05 mmol, 0.024 g, 10 mol%), in 1:1 DMSO/dioxane (5 mL). After 16 hours the reaction was quenched with 1M HCl (10 mL), extracted three times with ethyl acetate, dried over $MgSO_4$ and concentrated *in vacuo*. The crude reaction mixture was purified by silica chromatography purified by silica chromatography (0-50% EtOAc/hexanes + 1% AcOH followed by 0-10% MeOH/CH_2Cl_2 as the eluent) to afford the title compound as a white solid (0.058 g, 84% yield). The physical and spectral properties were consistent with the reported values.[15]
^{1}H NMR (400 MHz, DMSO-d_6) δ 13.30 (bs, 1H), 7.80 (dt, J = 7.6, 1.3 Hz, 1H), 7.69-7.63 (m, 1H), 7.61-7.54 (m, 1H), 7.53-7.46 (m, 1H).

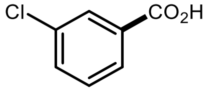

3-chlorobenzoic acid (16): Prepared according to the general procedure using 1-bromo-3-chlorobenzene (0.5 mmol, 59 µl, 1 equiv.), sodium formate (0.75 mmol, 0.051 g, 1.5 equiv.), 4CzIPN (.005 mmol, 0.0039 g, 1 mol%), N-phenyl-bis(trifluoromethanesulfonimide) (0.075 mmol, 0.027 g, 15 mol%), and [4,4'-Bis(1,1-dimethyl)-2,2'-bipyridine] nickel (II) bromide (0.05 mmol, 0.024 g, 10 mol%), in 1:1 DMSO/dioxane (5 mL). After 16 hours the reaction was quenched with 1M HCl (10 mL), extracted three times with ethyl acetate, dried over $MgSO_4$ and concentrated *in vacuo*. The crude reaction mixture was purified by silica chromatography purified by silica chromatography (0-50% EtOAc/hexanes + 1% AcOH followed by 0-10% MeOH/CH2Cl_2 as the eluent) to afford the title compound as a white solid (0.065 g, 83% yield). The physical and spectral properties were consistent with the reported values.[16]
^{1}H NMR (400 MHz, DMSO-d_6) δ 13.34 (bs, 1H), 7.93-7.89 (m, 2H), 7.74-7.69 (m, 1H), 7.55 (t, J = 8.1 Hz, 1H).

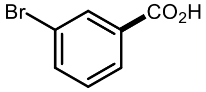

3-bromobenzoic acid (17): Prepared according to the general procedure using 1-bromo-3-iodobenzene (0.5 mmol, 64 µl, 1 equiv.), sodium formate (0.75 mmol, 0.051 g, 1.5 equiv.), 4CzIPN (.005 mmol, 0.0039 g, 1 mol%), N-phenyl-bis(trifluoromethanesulfonimide) (0.075 mmol, 0.027 g, 15 mol%), and [4,4'-Bis(1,1-dimethyl)-2,2'-bipyridine] nickel (II) bromide (0.05 mmol, 0.024 g, 10 mol%), in 1:1 DMSO/dioxane (5 mL). After 16 hours the reaction was quenched with 1M HCl (10 mL), extracted three times with ethyl acetate, dried over $MgSO_4$ and concentrated *in vacuo*. The crude reaction mixture was purified by silica chromatography purified by silica chromatography (10-20% EtOAc/hexanes + 1% AcOH as the eluent) to afford the title compound as a light yellow solid (0.082 g, 82% yield). The physical and spectral properties were consistent with the reported values.[17]
^{1}H NMR (400 MHz, DMSO-d$_6$) δ 13.32 (bs, 1H), 8.04 (t, J = 1.8 Hz, 1H), 7.93 (dt, J = 7.8, 1.3 Hz, 1H), 7.86-7.82 (m, 1H), 7.47 (t, J = 7.9 Hz).

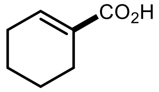

Cyclohex-1-ene-1-carboxylic acid (18): Prepared according to the general procedure **S3** (0.1 mmol, 12 µl, 1 equiv.), sodium formate (0.15 mmol, 0.010 g, 1.5 equiv.), 4CzIPN (.0001 mmol, 0.0008 g, 1 mol%), N-phenyl-bis(trifluoromethanesulfonimide) (0.015 mmol, 0.0053 g, 15 mol%), and [4,4'-Bis(1,1-dimethyl)-2,2'-bipyridine] nickel (II) bromide (0.01 mmol, 0.0048 g, 10 mol%), in 1:1 DMSO/dioxane (5 mL). After 16 hours the reaction was quenched with 1M HCl (10 mL), extracted three times with ethyl acetate, dried over $MgSO_4$ and concentrated *in vacuo*. Dibromomethane (0.1 mmol, 7.0 µl, 1.0 equiv.) was added to the crude reaction mixture as an internal standard and the sample was analyzed via ^{1}H NMR (d = 5 s), and the integral values were used to calculate product yield (80% yield by NMR).[18]
Note: Vinyl bromides suffered from issues with scalability/purification in this transformation, as such this reaction was performed on a 0.1 mmol scale and yield was determined via ^{1}H NMR using CH_2Br_2 as an internal standard..
^{1}H NMR (400 MHz, DMSO-d$_6$) δ 12.06 (s, 1H), 6.86 (m, 1H), 2.17-2.09 (m, 4H), 1.61-1.47 (m, 4H).

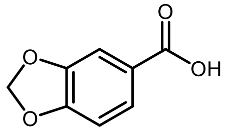

Benzo[*d*][1,3]dioxole-5-carboxylic acid (19): Prepared according to the general procedure using 5-bromo-1,3-benzodioxole (0.5 mmol, 60 µl, 1 equiv.), sodium formate (0.75 mmol, 0.051 g, 1.5 equiv.), 4CzIPN (.005 mmol, 0.0039 g, 1 mol%), N-phenyl-bis(trifluoromethanesulfonimide) (0.075 mmol, 0.027 g, 15 mol%), and [4,4'-Bis(1,1-dimethyl)-2,2'-bipyridine] nickel (II) bromide (0.05 mmol, 0.024 g, 10 mol%), in 1:1 DMSO/dioxane (5 mL). After 16 hours the reaction was quenched with 1M HCl (10 mL), extracted three times with ethyl acetate, dried over $MgSO_4$ and concentrated *in vacuo*. The crude reaction mixture was purified by silica chromatography purified by silica chromatography (20-30% EtOAc/hexanes + 1% AcOH as the eluent) to afford the title compound as a white solid (0.055 g, 66% yield). The physical and spectral properties were consistent with the reported values.[19]

^{1}H NMR (400 MHz, DMSO-d$_6$) δ 12.76 (s, 1H), 7.54 (dd, J = 8.3, 1.7 Hz, 1H), 7.36 (d, J = 1.7 Hz, 1H), 7.00 (d, J = 8.1 Hz, 1H), 6.12 (s, 2H).

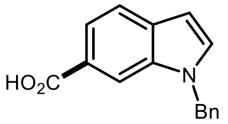

1-benzyl-1*H*-indole-6-carboxylic acid (20): Prepared according to the general procedure using 1-benzyl-6-bromo-1*H*-indole (0.5 mmol, 0.143 g, 1 equiv.), sodium formate (0.75 mmol, 0.051 g, 1.5 equiv.), 4CzIPN (.005 mmol, 0.0039 g, 1 mol%), N-phenyl-bis(trifluoromethanesulfonimide) (0.075 mmol, 0.027 g, 15 mol%), and [4,4'-Bis(1,1-dimethyl)-2,2'-bipyridine] nickel (II) bromide (0.05 mmol, 0.024 g, 10 mol%), in 1:1 DMSO/dioxane (5 mL). After 16 hours the reaction was quenched with 1M HCl (10 mL), extracted three times with ethyl acetate, dried over $MgSO_4$ and concentrated *in vacuo*. The crude reaction mixture was purified by silica chromatography purified by silica chromatography (10-30% EtOAc/hexanes + 1% AcOH as the eluent) to afford the title compound as a light yellow solid (0.063 g, 50% yield). The physical and spectral properties were consistent with the reported values.[1]
^{1}H NMR (400 MHz, DMSO-d$_6$) δ 12.57 (bs, 1H), 8.05-8.04 (m, 1H), 7.77 (d, J = 3.0 Hz, 1H), 7.63-7.61 (m, 2H), 7.31 (t, J = 7.1 Hz, 2H), 7.25 (t, J = 7.4 Hz, 1H), 7.16 (d, J = 7.4 Hz, 2H), 6.59 (dd, J = 3.1, 0.9 Hz, 1H), 5.53 (s, 2H).
^{13}C {^{1}H} NMR (100 MHz, DMSO-d$_6$) δ 168.2, 138.1, 135.1, 132.8, 131.8, 128.6, 127.4, 126.7, 123.5, 120.2, 120.1, 112.2, 101.4, 49.2.
HRMS (APCI) *m/z*: [M+H] calcd. for $C_{16}H_{14}O_2N$, 252.1019, found 252.1015.
R$_f$: 0.49 (30% EtOAc/Hexanes + 1% AcOH)

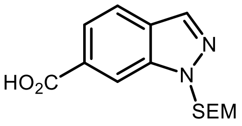

1-((2-(trimethylsilyl)ethoxy)methyl)-1*H*-indazole-6-carboxylic acid (21): Prepared according to the general procedure using **S5** (0.5 mmol, 0.143 g, 1 equiv.), sodium formate (0.75 mmol, 0.051 g, 1.5 equiv.), 4CzIPN (.005 mmol, 0.0039 g, 1 mol%), N-phenyl-bis(trifluoromethanesulfonimide) (0.075 mmol, 0.027 g, 15 mol%), and [4,4'-Bis(1,1-dimethyl)-2,2'-bipyridine] nickel (II) bromide (0.05 mmol, 0.024 g, 10 mol%), in 1:1 DMSO/dioxane (5 mL). After 16 hours the reaction was quenched with 1M HCl (10 mL), extracted three times with ethyl acetate, dried over $MgSO_4$ and concentrated *in vacuo*. The crude reaction mixture was purified by silica chromatography purified by silica chromatography (10-30% EtOAc/hexanes + 1% AcOH as the eluent) to afford the title compound as a light yellow solid (0.066 g, 66% yield). The physical and spectral properties were consistent with the reported values.[1]
^{1}H NMR (400 MHz, DMSO-d$_6$) δ 13.13 (bs, 1H), 8.35 (d, J = 1.1 Hz, 1H), 8.25 (d, J = 1.0 Hz, 1H), 7.89 (dd, J = 8.5, 0.8Hz, 1H), 7.75 (dd, J = 8.4, 1.3 Hz, 1H), 5.85 (s, 2H), 3.51 (t, J = 8.1 Hz, 2H), 0.79 (t, J = 7.9 Hz, 2H), -0.13 (s, 9H).
^{13}C {^{1}H} NMR (100 MHz, DMSO-d$_6$) δ 167.5, 139.2, 134.1, 128.9, 126.7, 121.5, 121.0, 112.0, 76.9, 65.6, 17.1, -1.2, -1.4, -1.7.
HRMS (APCI) *m/z*: [M+H] calcd. for $C_{14}H_{21}O_3N_2Si$, 293.1316, found 293.1313.

R_f: 0.52 (30% EtOAc/Hexanes + 1% AcOH)

VIII. References

1. Roth, G.; Romero, N.; Nicewicz, D. Experimental and Calculated Electrochemical Potentials of Common Organic Molecules for Applications to Single-Electron Redox Chemistry. *Synlett.*, **2017**, *5*, 714-723.
2. Shi, S.; Lalancette, R.; Szostak, R.; Szostak, M. Triflamides: Highly Reactive, Electronically Activated N-Sulfonyl Amides in Catalytic N-C(O) Amide Cross-Coupling. *Org. Lett*., **2019**, *21*, 1253-1257.
3. Grotjahn, S.; Konig, B. Photosubstitution in Dicyanobenzene-based Photocatalysts. *Org. Lett*., **2021**, *23*, 3146-3150.
4**.** Ojha, D. P., & Prabhu, K. R. . Regioselective Synthesis of Vinyl Halides, Vinyl Sulfones, and Alkynes: A Tandem Intermolecular Nucleophilic and Electrophilic Vinylation of Tosylhydrazones. *Org. Lett.*, **2015**, *17*, 18–21.
5. Zhang, X. L., Guo, R. L., Wang, M. Y., Zhao, B. Y., Jia, Q., Yang, J. H., & Wang, Y. Q. .Palladium-Catalyzed Three-Component Regioselective Dehydrogenative Coupling of Indoles, 2-Methylbut-2-ene, and Carboxylic Acids. *Org. Lett.,* **2021**, *23*, 9574–9579.
6. Tang, S., Rauch, M., Montag, M., Diskin-Posner, Y., Ben-David, Y., & Milstein, D. Catalytic Oxidative Deamination by Water with H2 Liberation. *J. Am. Chem. Soc*., **2020**, *142*, 20875-20882.
7. Farizyan, M., Mondal, A., Mal, S., Deufel, F., & van Gemmeren, M. Palladium-Catalyzed Nondirected Late-Stage C-H Deuteration of Arenes. *J. Am. Chem. Soc.*, **2021**, *143*, 16370–16376.
8. Kim, S. M., Kim, D. W., & Yang, J. W. Transition-metal-free and Chemoselective NaOtBu-O 2-mediated oxidative cleavage reactions of vic-1,2-diols to carboxylic acids and mechanistic insight into the reaction pathways. *Org. Lett.*, **2014**, 16, 2876–2879.
9. Wang, Y., Zhao, Z., Pan, D., Wang, S., Jia, K., Ma, D., Yang, G., Xue, X. S., & Qiu, Y. Metal-Free Electrochemical Carboxylation of Organic Halides in the Presence of Catalytic Amounts of an Organomediator. *Angew. Chem. Int. Ed.*, **2022**, 61, e202210201.
10. Ma, C., Zhao, C. Q., Xu, X. T., Li, Z. M., Wang, X. Y., Zhang, K., & Mei, T. S. Nickel-catalyzed carboxylation of aryl and heteroaryl fluorosulfates using carbon dioxide. *Org. Lett.*, **2019**, *21*, 2464–2467.
11. (a) Peixoto, D.; Figueiredo, M.; Gawande, M.; Corvo, M.; Vanhoenacker, G.; Afonso, C.; Ferreira, L.; Branco, P. Developments in the Reactivity of 2-Methylimidazolium Salts. *J. Org. Chem.*, **2017**, *82*, 6232-6241.
(b) Bhunia, S.; Das, P.; Nandi, S.; Jana, R. Carboxylation of Aryl Triflates with CO2 Merging Palladium and Visible-Light-Photoredox Catalysts. *Org. Lett*., **2019**, *21*, 4632-4737.
12. Liu, W., Wang, H., & Li, C. J. Metal-Free Markovnikov-Type Alkyne Hydration under Mild Conditions. *Org. Lett.*, **2016**, *18*, 2184–2187.
13. Wise, D. E., Gogarnoiu, E. S., Duke, A. D., Paolillo, J. M., Vacala, T. L., Hussain, W. A., & Parasram, M. Photoinduced Oxygen Transfer Using Nitroarenes for the Anaerobic Cleavage of Alkenes. *J. Am. Chem. Soc.*, **2022**, *144*, 15437–15442.
14. Weidel, E., de Jong, J. C., Brengel, C., Storz, M. P., Braunshausen, A., Negri, M., Plaza, A., Steinbach, A., Müller, R., & Hartmann, R. W. Structure optimization of 2-benzamidobenzoic

acids as PqsD inhibitors for Pseudomonas aeruginosa infections and elucidation of binding mode by SPR, STD NMR, and molecular docking. *J. Med. Chem.*, **2013**, *56*, 6146–6155.
15. Xia, A., Qi, X., Mao, X., Wu, X., Yang, X., Zhang, R., Xiang, Z., Lian, Z., Chen, Y., & Yang, S. Metal-Free Aerobic Oxidative Selective C-C Bond Cleavage in Heteroaryl-Containing Primary and Secondary Alcohols. *Org. Lett.*, **2019**, *2*, 3028–3033.
16. Zhang, Z., Zhang, G., Xiong, N., Xue, T., Zhang, J., Bai, L., Guo, Q., & Zeng, R. Oxidative α-C-C Bond Cleavage of 2° and 3° Alcohols to Aromatic Acids with O2at Room Temperature via Iron Photocatalysis. *Org. Lett.*, **2021**, *23*, 2915–2920.
17. Wang, W., Yang, X., Dai, R., Yan, Z., Wei, J., Dou, X., Qiu, X., Zhang, H., Wang, C., Liu, Y., Song, S., & Jiao, N. Catalytic Electrophilic Halogenation of Arenes with Electron-Withdrawing Substituents. *J. Am. Chem. Soc.*, **2022**, *144*, 13415–13425.
18. Monda, F., & Madsen, R. Zinc Oxide-Catalyzed Dehydrogenation of Primary Alcohols into Carboxylic Acids. *Chem. Eur. J.*, **2018**, *24*, 17832–17837.
19. Ou, J., Tan, H., He, S., Wang, W., Hu, B., Yu, G., & Liu, K. 1,2-Dibutoxyethane-Promoted Oxidative Cleavage of Olefins into Carboxylic Acids Using O2 under Clean Conditions. *J. Org. Chem.*, **2021**, *86*, 14974–14982.
20. (a) Ai, Y.; Ye, N.; Wang, Q.; Yahata, K.; Kishi, Y. Zirconium/Nickel-Mediated One-Pot Ketone Synthesis. *Angew. Chem. Int. Ed.*, **2017**, 56, 10791-10795.
(b) Ren, H.; Li, G.; Zhu, B.; Lv, X.; Yao, L.; Wang, X.; Su, Z.; Guan, W. How Does Iridium (III) Photocatalyst Regulate Nickel (II) Catalyst in Metallaphotoredox-Catalyzed C-S Cross-Coupling? Theoretical and Experimental Insights. *ACS. Catal*., **2019**, *9*, 5, 3858-3865.
21. (a) Shi, S.; Lalancette, R.; Szostak, R.; Szostak, M. Triflamides: Highly Reactive, Electronically Activated N-Sulfonyl Amides in Catalytic N-C(O) Amide Cross-Coupling. *Org. Lett*., **2019**, *21*, 1253-1257.
(b) Ball-Jones, N.; Badillo, J.; Tran, N.; Franz, A. Catalytic Enantioselective Carboannulation with Allylsilanes. *Angew. Chem. Int. Ed*., **2014**, *53*, 9462-9465.
22. Hendy, C.; Smith, G.; Xu, Z.; Lian, T.; Jui, N. Radical Chain Reduction via Carbon Dioxide Radical Anion ($CO_2^{\bullet-}$). *J. Am. Chem. Soc*., **2021**, *143*, 8987-8992.

IX. NMR Spectra

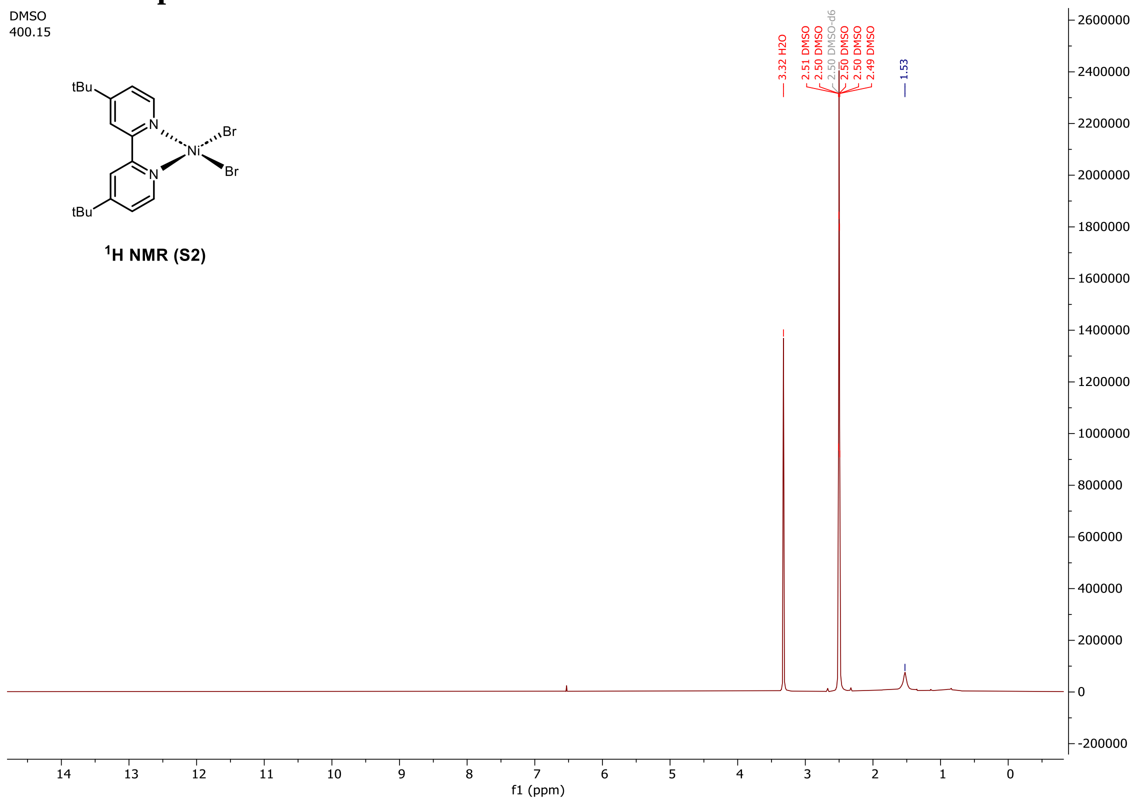

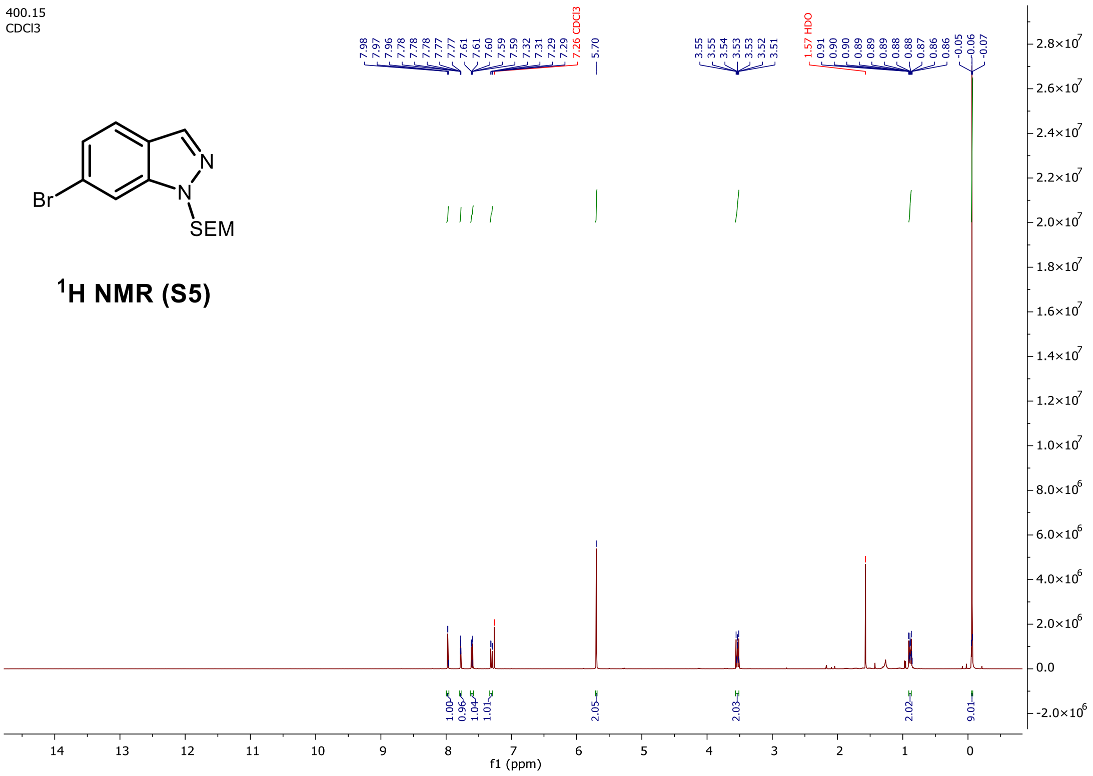

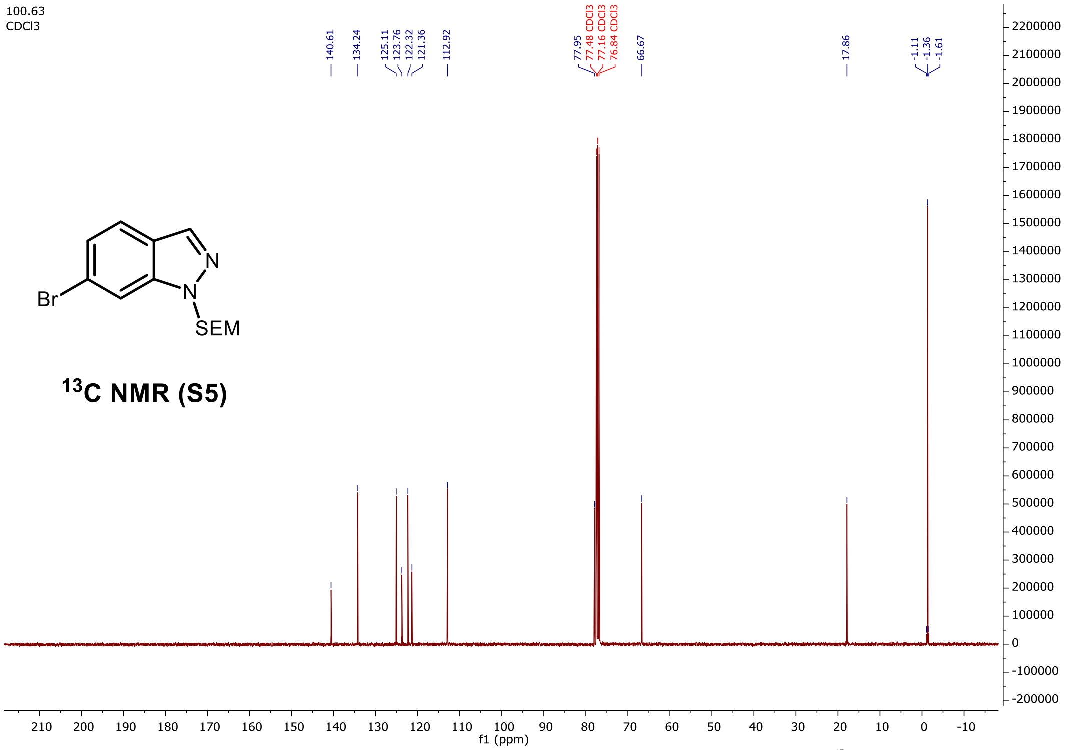
100.63
CDCl3
140.61
134.24
125.11
123.76
122.32
121.36
112.92
77.95
77.48 CDCl3
77.16 CDCl3
76.84 CDCl3
66.67
17.86
-1.11
-1.36
-1.61
Br
N
N
SEM
13C NMR (S5)
f1 (ppm)

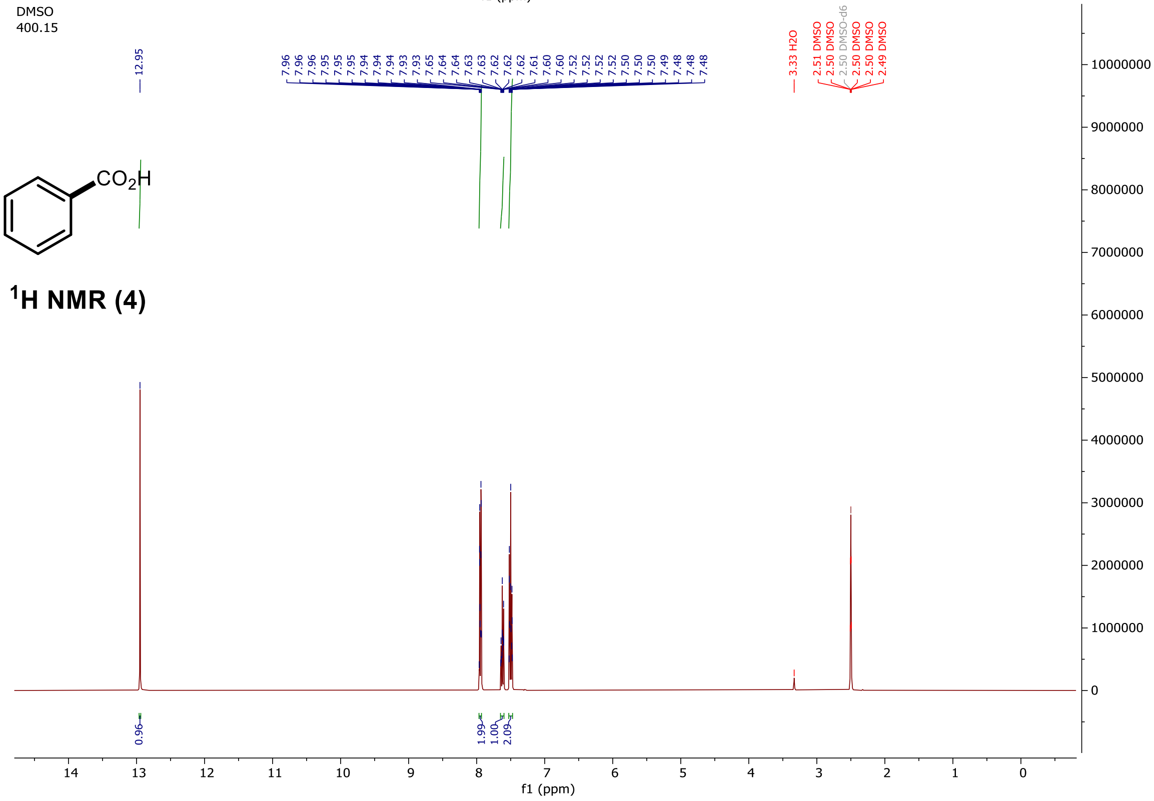
DMSO
400.15
12.95
7.96
7.96
7.96
7.95
7.95
7.95
7.94
7.94
7.94
7.93
7.93
7.65
7.64
7.64
7.63
7.63
7.62
7.62
7.62
7.61
7.60
7.60
7.52
7.52
7.52
7.52
7.50
7.50
7.50
7.49
7.48
7.48
3.33 H2O
2.51 DMSO
2.50 DMSO
2.50 DMSO-d6
2.50 DMSO
2.50 DMSO
2.49 DMSO
CO2H
1H NMR (4)
0.96
1.99
1.00
2.09
f1 (ppm)

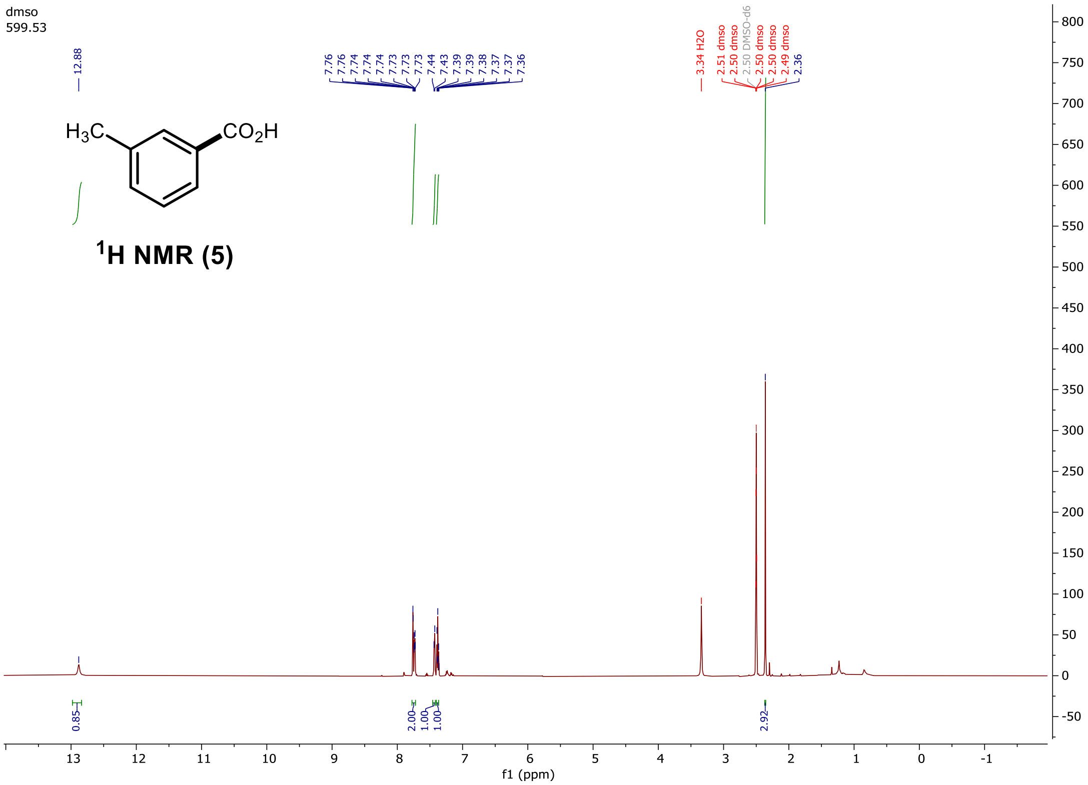
dmso
599.53
12.88
7.76 7.76 7.74 7.74 7.74 7.73 7.73 7.73 7.44 7.43 7.39 7.39 7.38 7.37 7.37 7.36
3.34 H2O
2.51 dmso 2.50 dmso 2.50 DMSO-d6 2.50 dmso 2.50 dmso 2.49 dmso 2.36
H3C CO2H
1H NMR (5)
0.85 2.00 1.00 1.00 2.92
f1 (ppm)

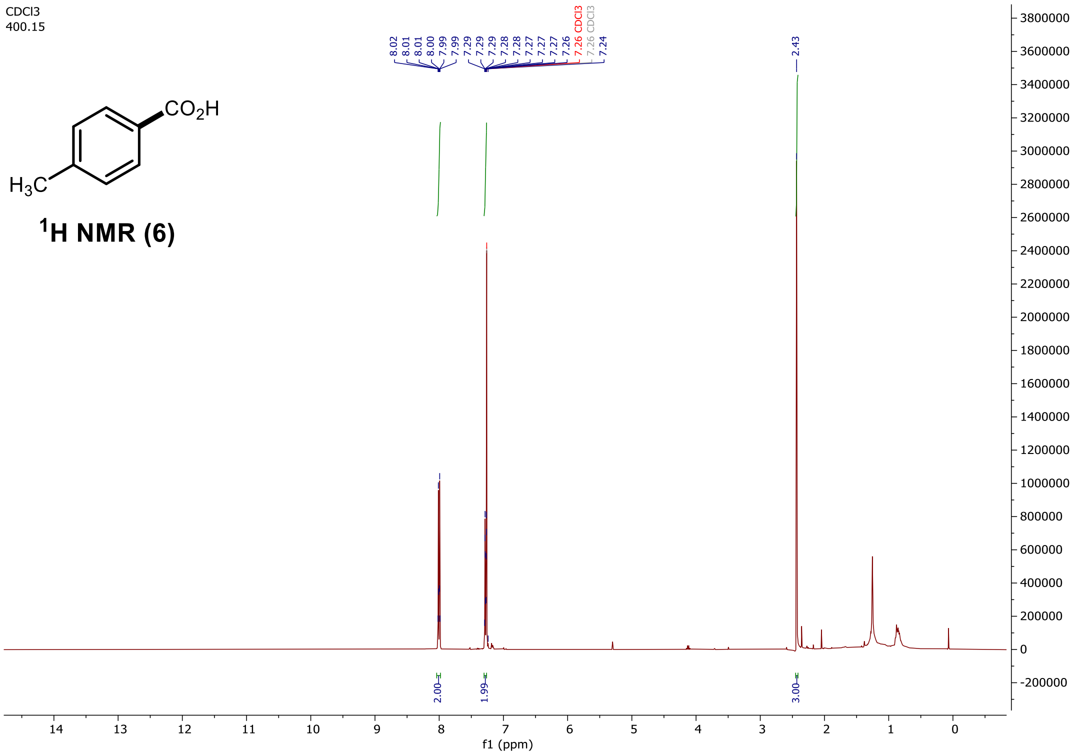
CDCl3
400.15
8.02 8.01 8.01 8.00 7.99 7.99 7.29 7.29 7.29 7.28 7.27 7.27 7.27 7.26 7.26 CDCl3 7.26 CDCl3 7.24
2.43
CO2H
H3C
1H NMR (6)
2.00 1.99 3.00
f1 (ppm)

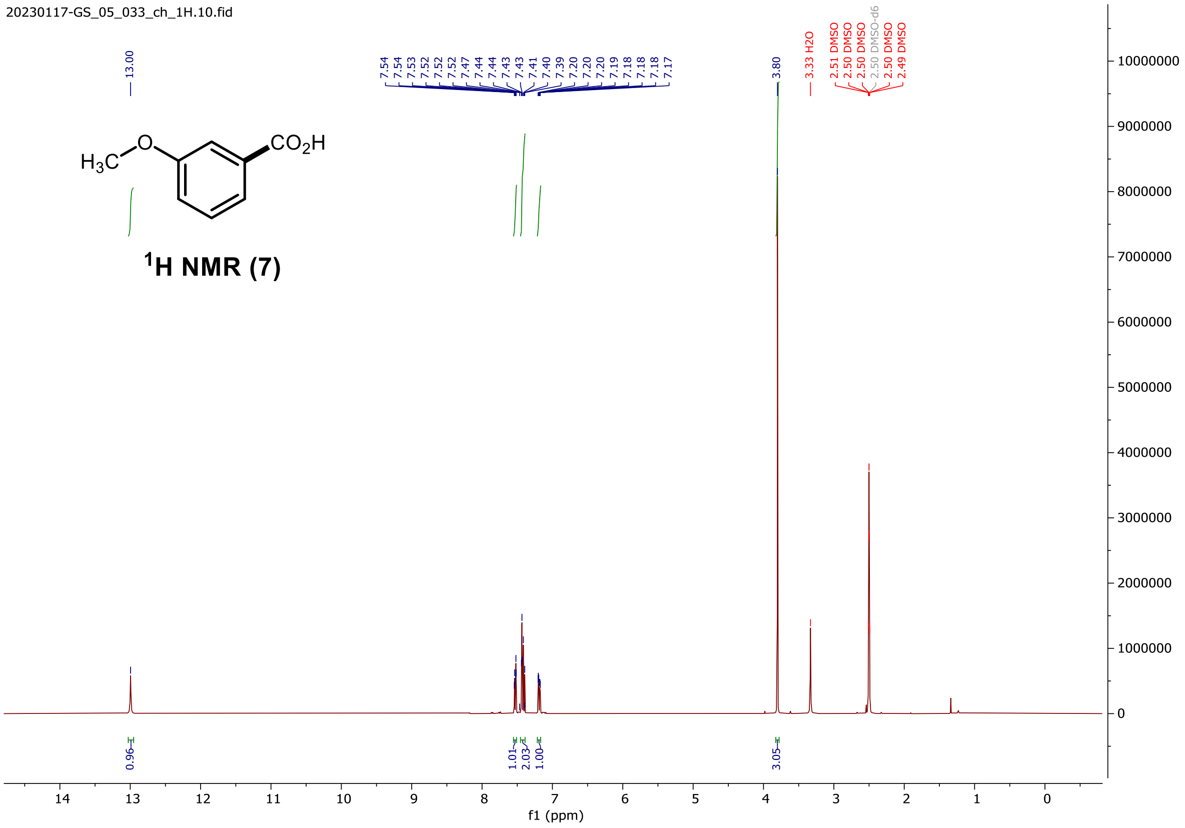
20230117-GS_05_033_ch_1H.10.fid
13.00
7.54 7.54 7.53 7.52 7.52 7.52 7.47 7.44 7.44 7.43 7.43 7.41 7.40 7.39 7.20 7.20 7.20 7.19 7.18 7.18 7.18 7.17
3.80
3.33 H2O
2.51 DMSO 2.50 DMSO 2.50 DMSO 2.50 DMSO-d6 2.50 DMSO 2.49 DMSO
H3C O CO2H
1H NMR (7)
0.96 1.01 2.03 1.00 3.05
f1 (ppm)

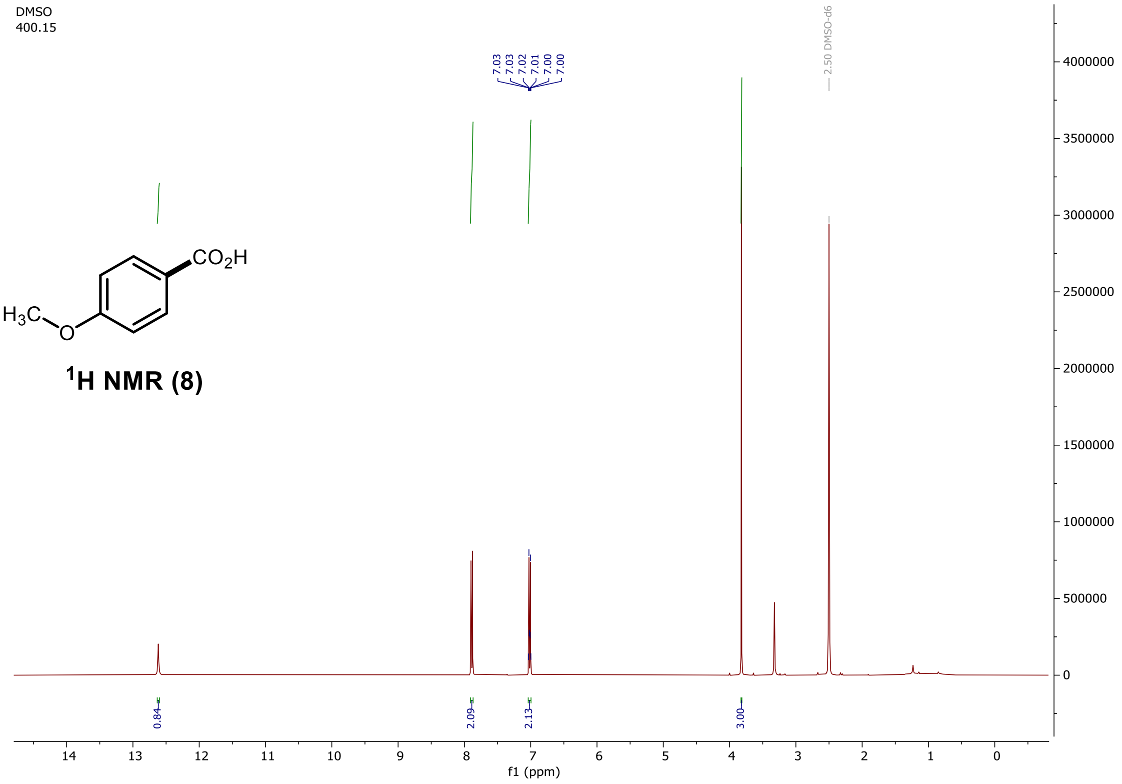
DMSO
400.15
7.03 7.03 7.02 7.01 7.00 7.00
2.50 DMSO-d6
CO2H H3C O
1H NMR (8)
0.84 2.09 2.13 3.00
f1 (ppm)

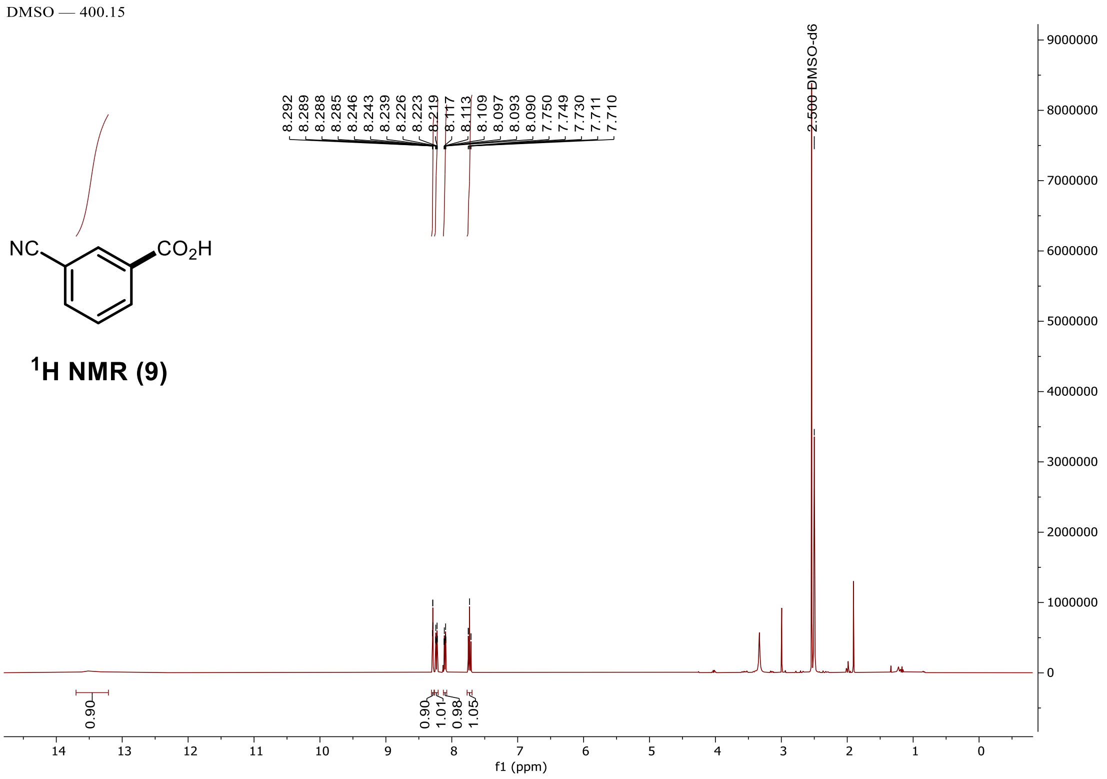
DMSO — 400.15
NC
CO2H
1H NMR (9)
2.500 DMSO-d6
f1 (ppm)

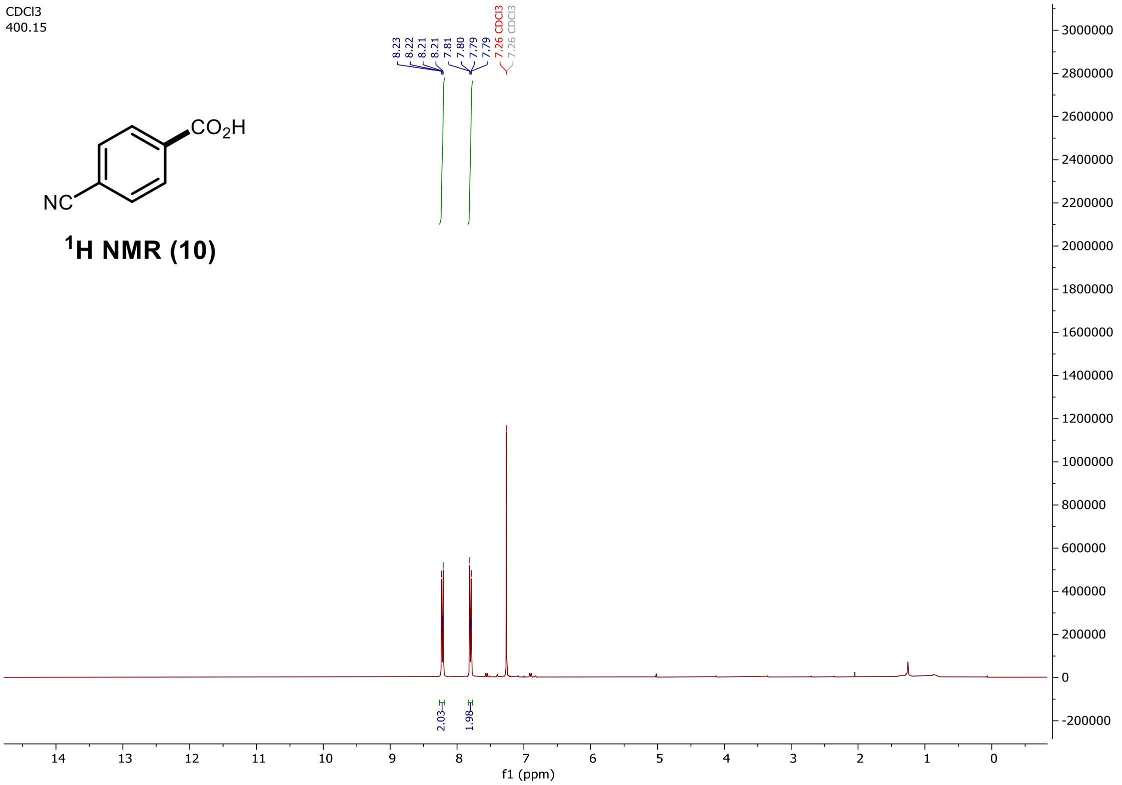
CDCl3
400.15
CO2H
NC
1H NMR (10)
f1 (ppm)

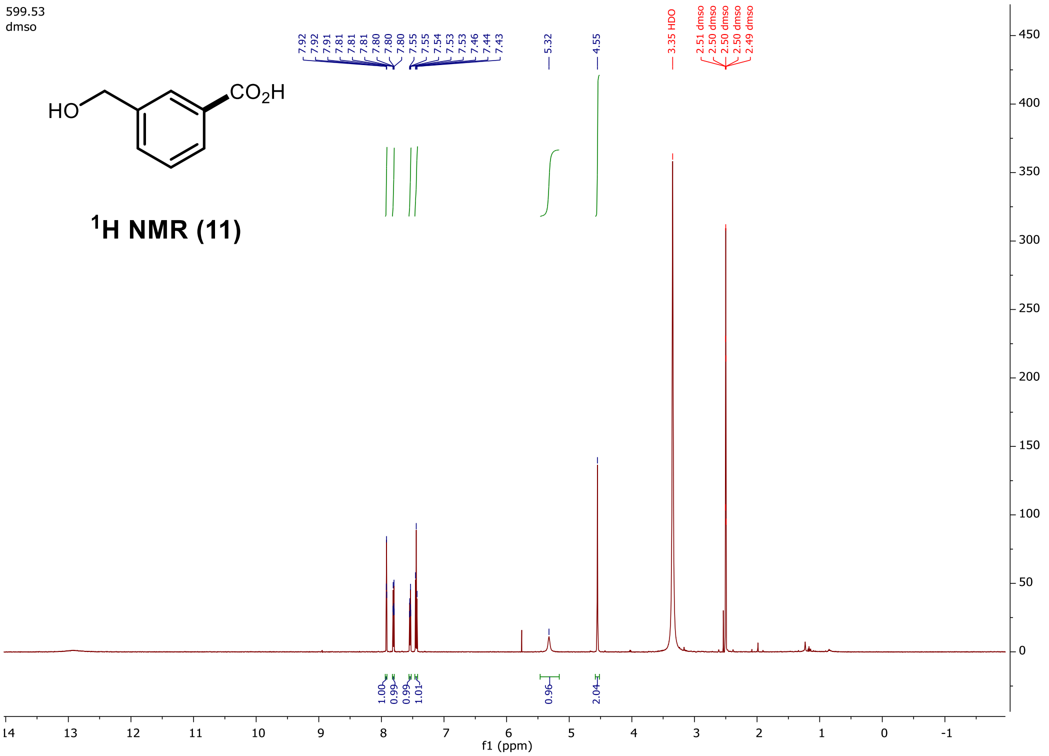

^{1}H NMR (11)

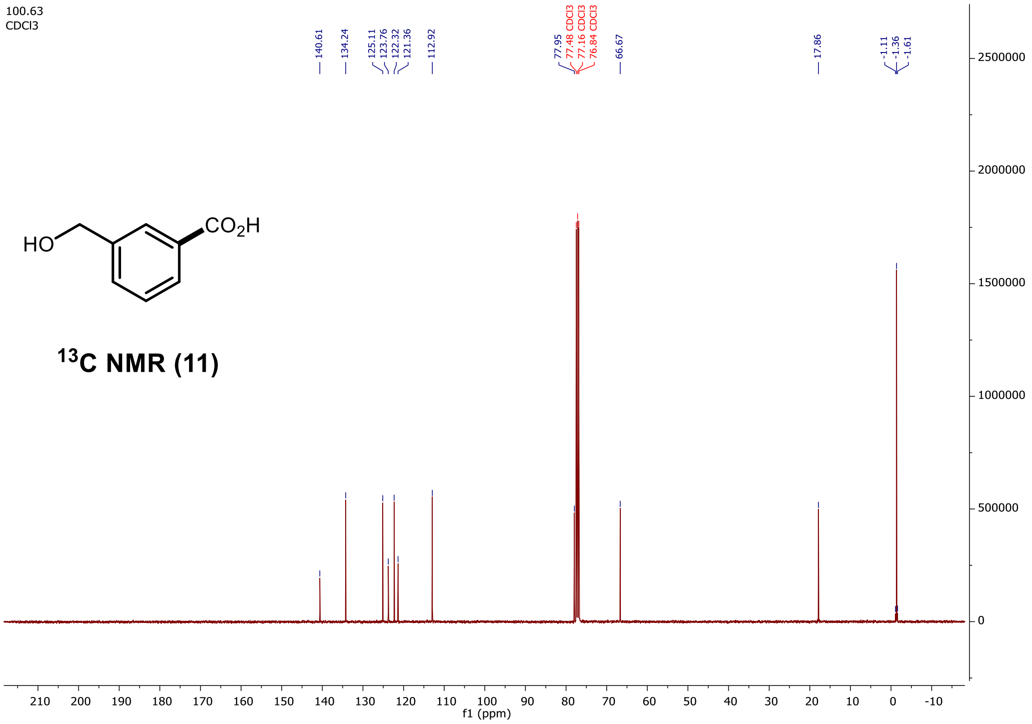

^{13}C NMR (11)

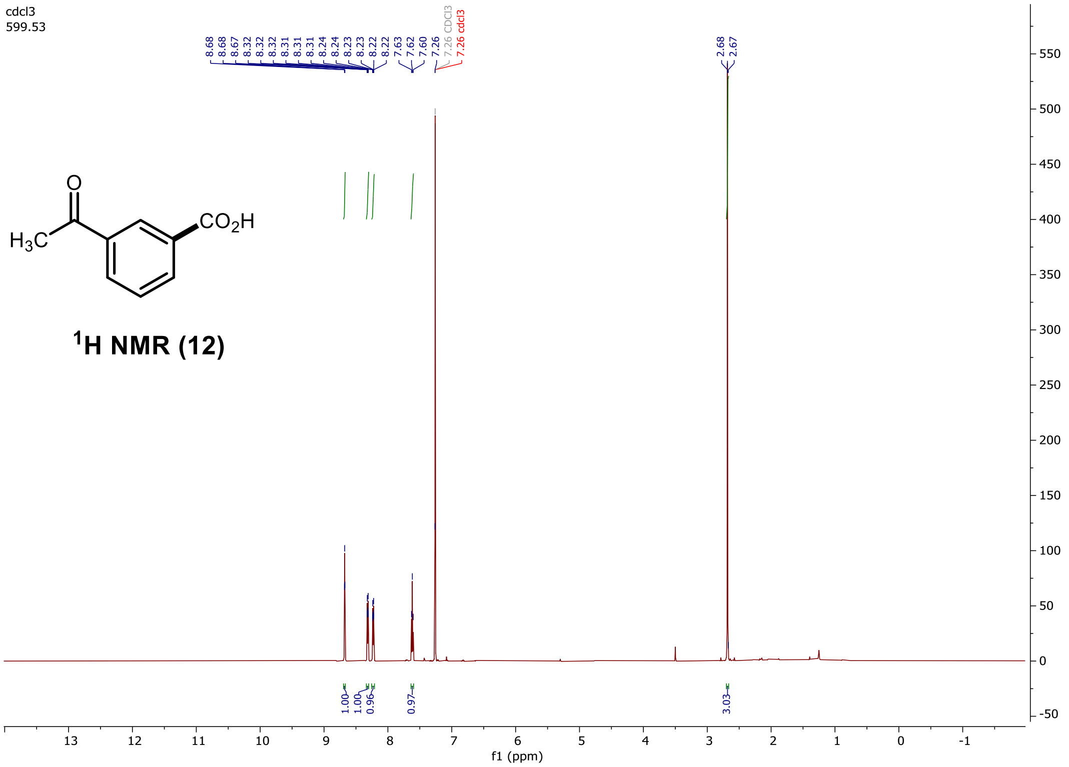

cdcl3
599.53
8.68
8.68
8.67
8.32
8.32
8.32
8.31
8.31
8.24
8.23
8.23
8.22
8.22
7.63
7.62
7.60
7.26 CDCl3
7.26 cdcl3
2.68
2.67
H3C
O
CO2H
1H NMR (12)
1.00
1.00
0.96
0.97
3.03
f1 (ppm)

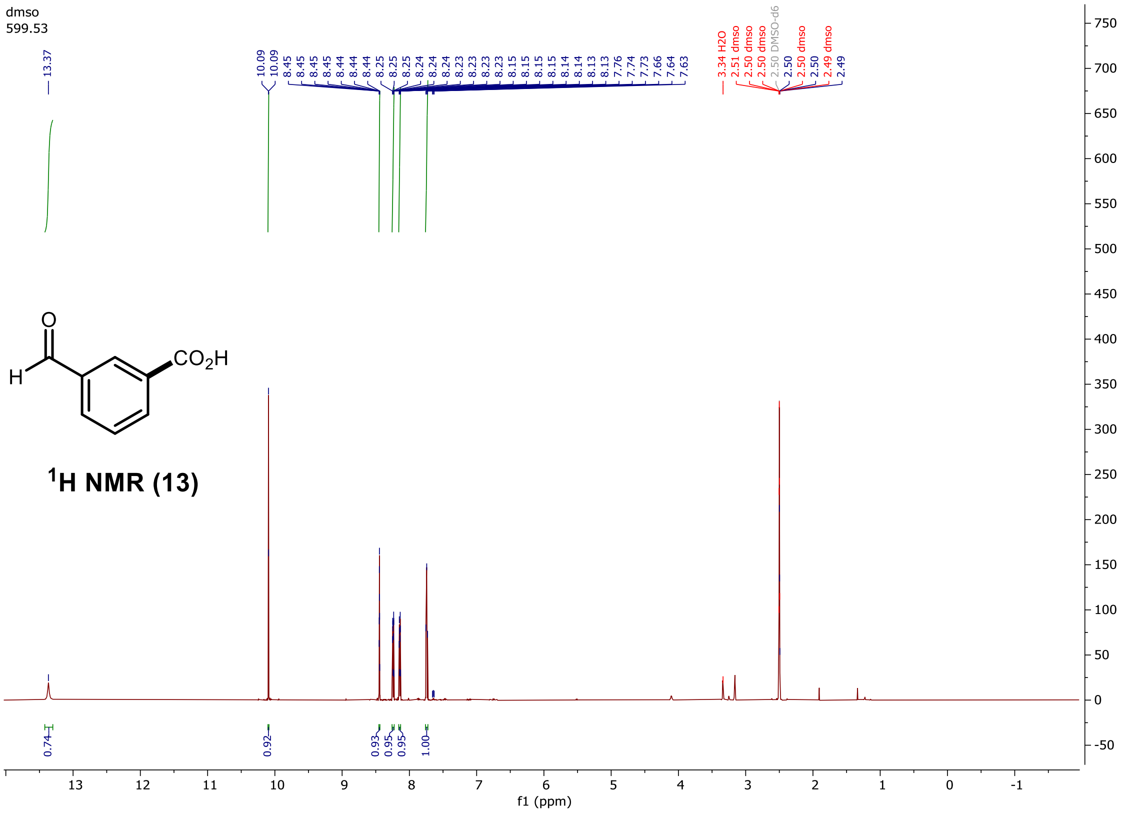
dmso
599.53
13.37
10.09
10.09
3.34 H2O
2.50 DMSO-d6
CO2H
1H NMR (13)
0.74
0.92
0.93
0.95
0.95
1.00
f1 (ppm)

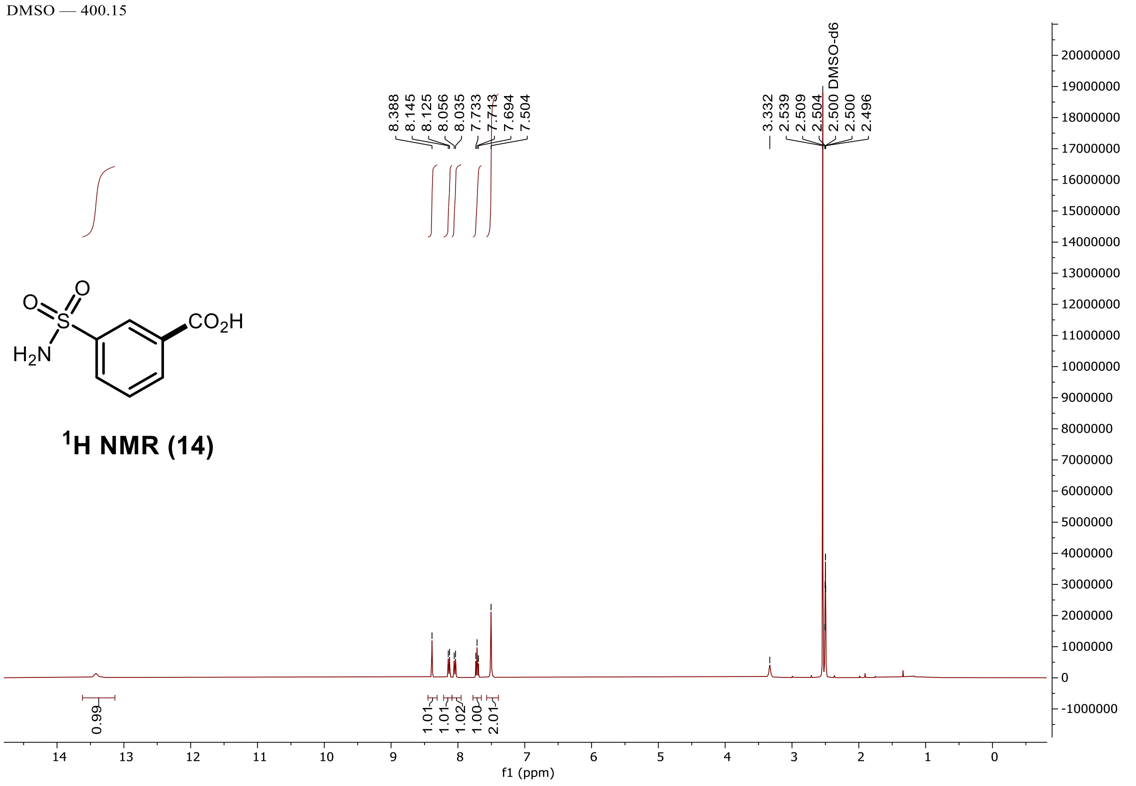
DMSO — 400.15
8.388
8.145
8.125
8.056
8.035
7.733
7.713
7.694
7.504
3.332
2.539
2.509
2.504
2.500 DMSO-d6
2.500
2.496
CO2H
H2N
1H NMR (14)
0.99
1.01
1.01
1.02
1.00
2.01
f1 (ppm)

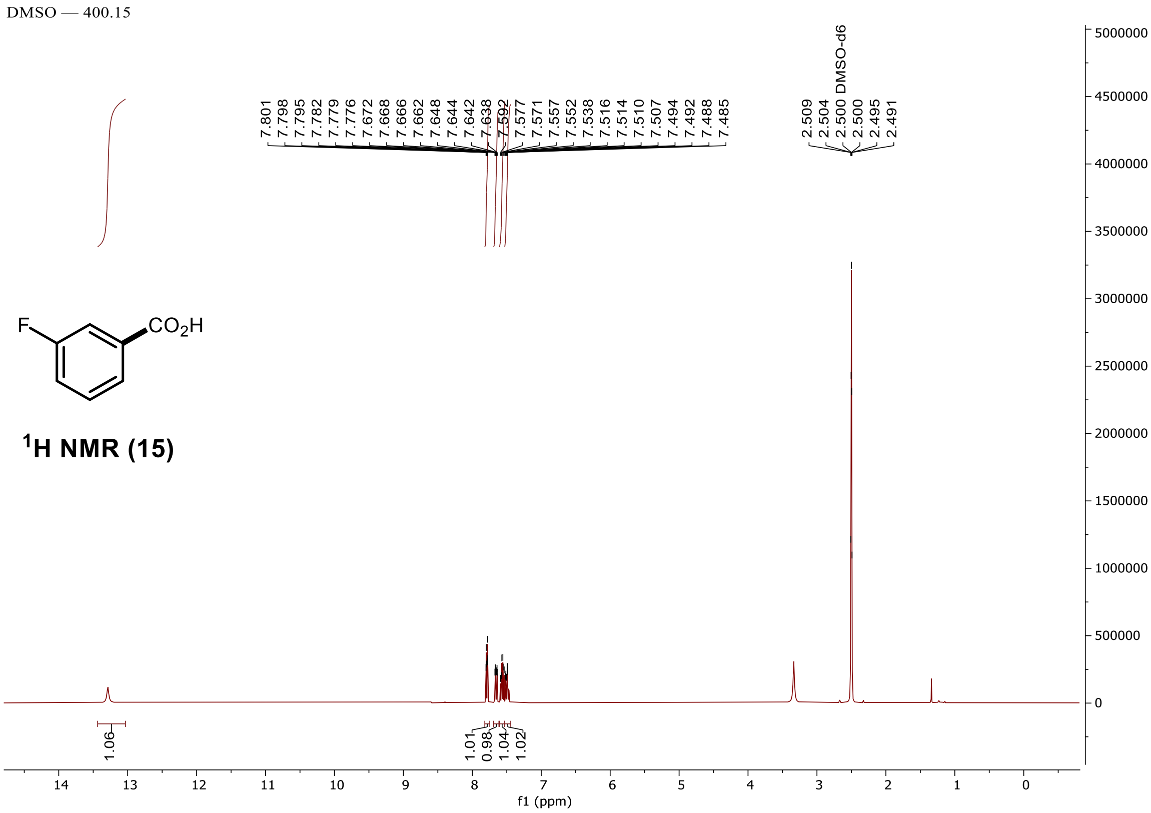
DMSO — 400.15
7.801
7.798
7.795
7.782
7.779
7.776
7.672
7.668
7.666
7.662
7.648
7.644
7.642
7.638
7.592
7.577
7.571
7.557
7.552
7.538
7.516
7.514
7.510
7.507
7.494
7.492
7.488
7.485
2.509
2.504
2.500 DMSO-d6
2.500
2.495
2.491
F
CO_2H
1H NMR (15)
1.06
1.01
0.98
1.04
1.02
f1 (ppm)

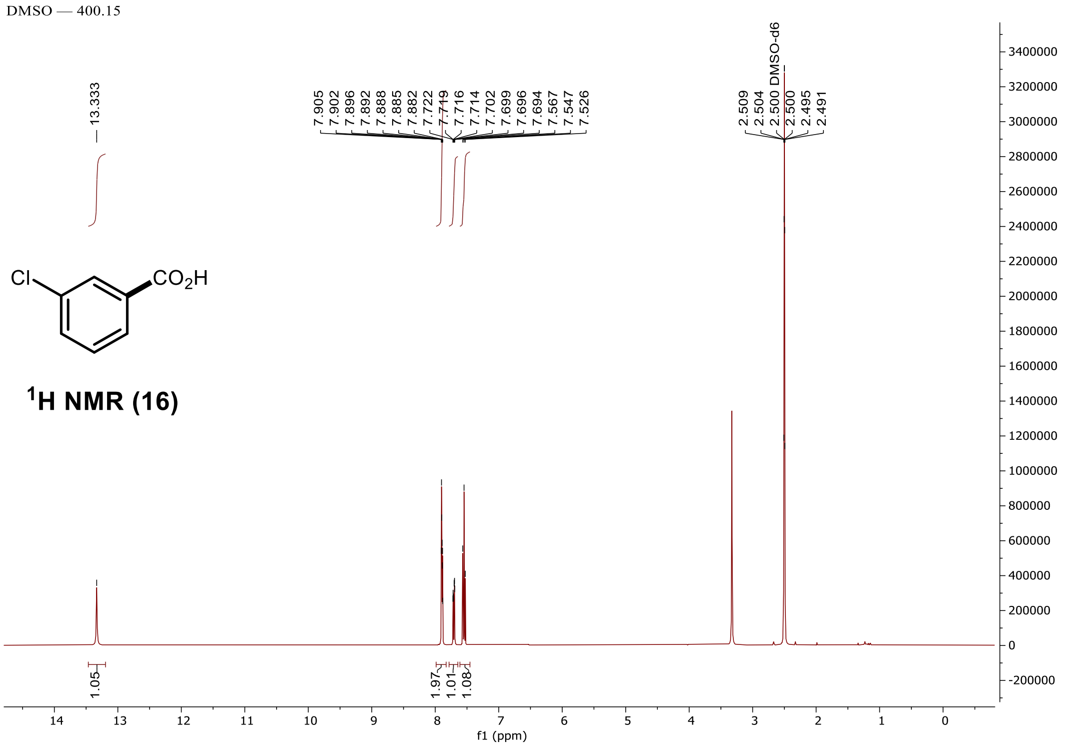
DMSO — 400.15
13.333
7.905
7.902
7.896
7.892
7.888
7.885
7.882
7.722
7.719
7.716
7.714
7.702
7.699
7.696
7.694
7.567
7.547
7.526
2.509
2.504
2.500 DMSO-d6
2.500
2.495
2.491
Cl
CO_2H
1H NMR (16)
1.05
1.97
1.01
1.08
f1 (ppm)

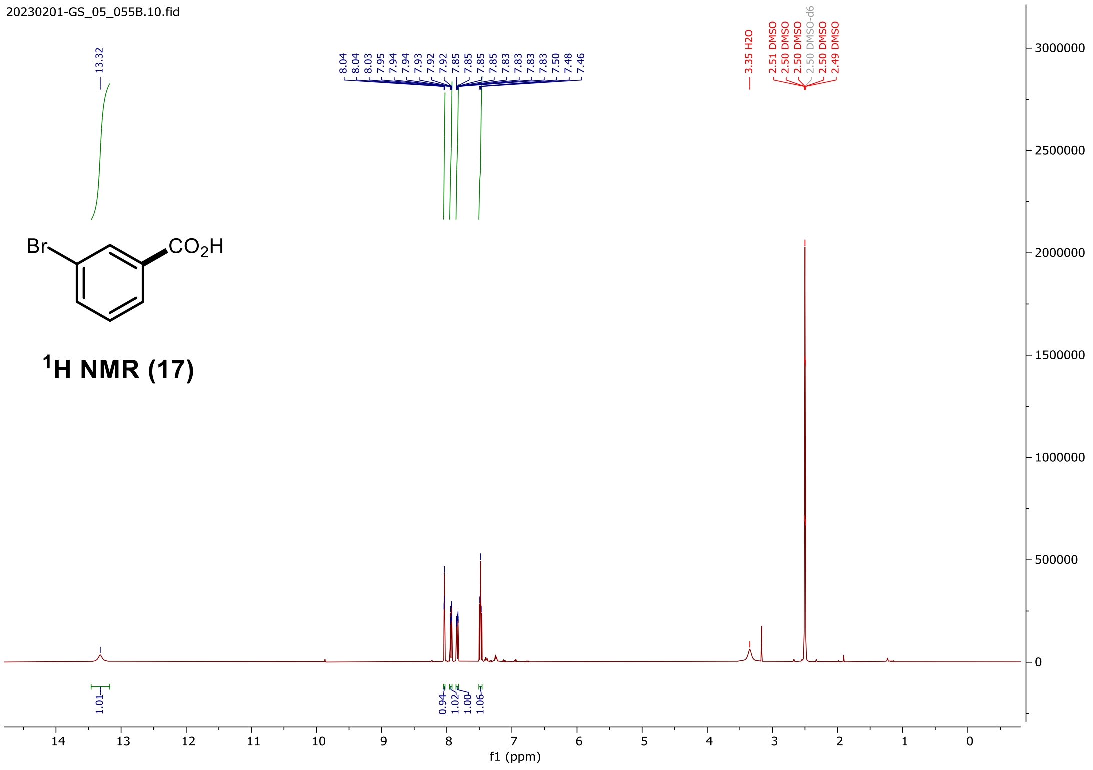
20230201-GS_05_055B.10.fid
13.32
8.04
8.04
8.03
7.95
7.94
7.94
7.93
7.92
7.92
7.85
7.85
7.85
7.85
7.83
7.83
7.83
7.83
7.50
7.48
7.46
3.35 H2O
2.51 DMSO
2.50 DMSO
2.50 DMSO
2.50 DMSO-d6
2.50 DMSO
2.49 DMSO
Br
CO_2H
^{1}H NMR (17)
1.01
0.94
1.02
1.00
1.06
f1 (ppm)

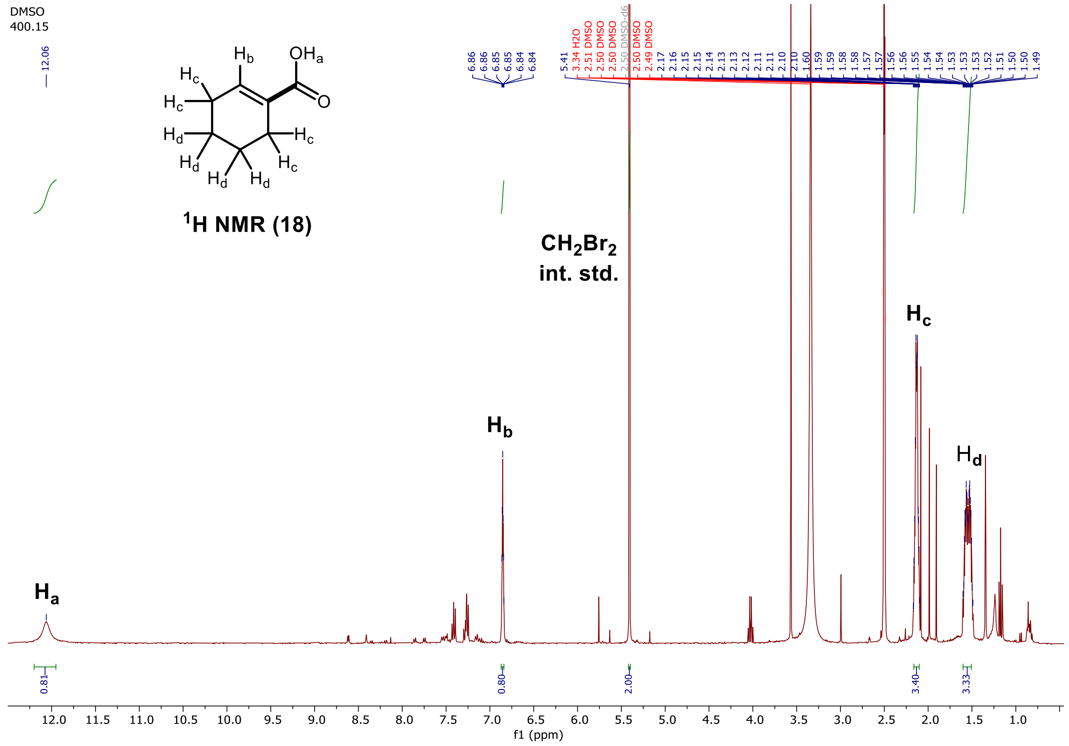
DMSO
400.15
12.06
H_b
OH_a
H_c
H_d
^{1}H NMR (18)
CH_2Br_2
int. std.
H_a
H_b
H_c
H_d
0.81
0.80
2.00
3.40
3.33
f1 (ppm)

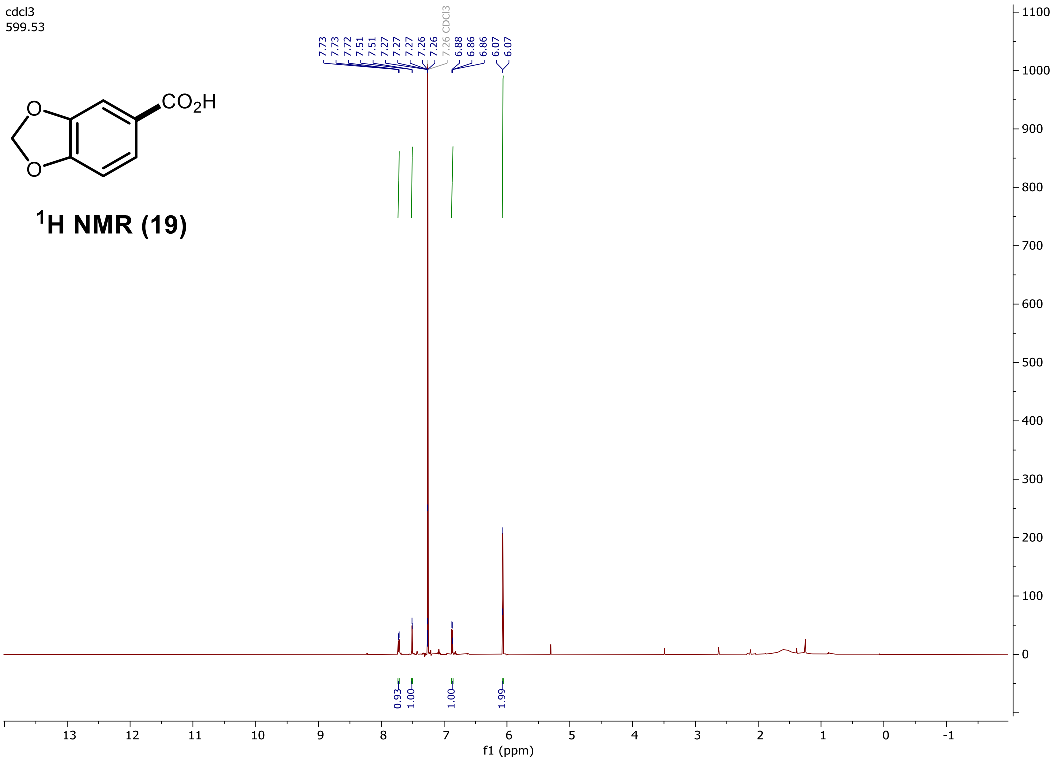
cdcl3
599.53
CO2H
O
O
1H NMR (19)
7.73
7.73
7.72
7.51
7.51
7.27
7.27
7.27
7.26
7.26
7.26 CDCl3
6.88
6.86
6.86
6.07
6.07
0.93
1.00
1.00
1.99
f1 (ppm)

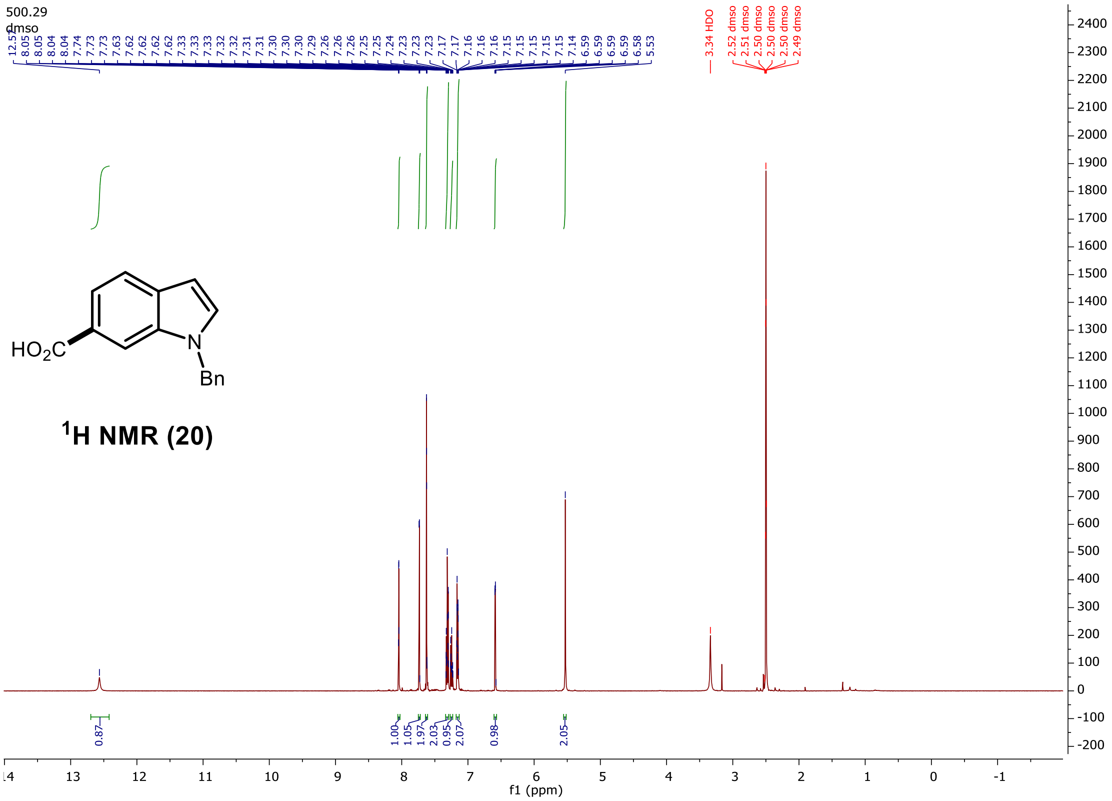
500.29
dmso
12.57 8.05 8.05 8.04 8.04 7.74 7.73 7.73 7.63 7.62 7.62 7.62 7.33 7.33 7.33 7.32 7.32 7.31 7.30 7.30 7.29 7.26 7.26 7.25 7.25 7.24 7.23 7.23 7.23 7.17 7.17 7.16 7.16 7.15 7.15 7.15 7.15 7.14 6.59 6.59 6.59 6.59 6.58 5.53
3.34 HDO
2.52 dmso 2.51 dmso 2.50 dmso 2.50 dmso 2.50 dmso 2.49 dmso
HO2C
N
Bn
1H NMR (20)
0.87 1.00 1.05 1.97 2.03 0.95 2.07 0.98 2.05
f1 (ppm)

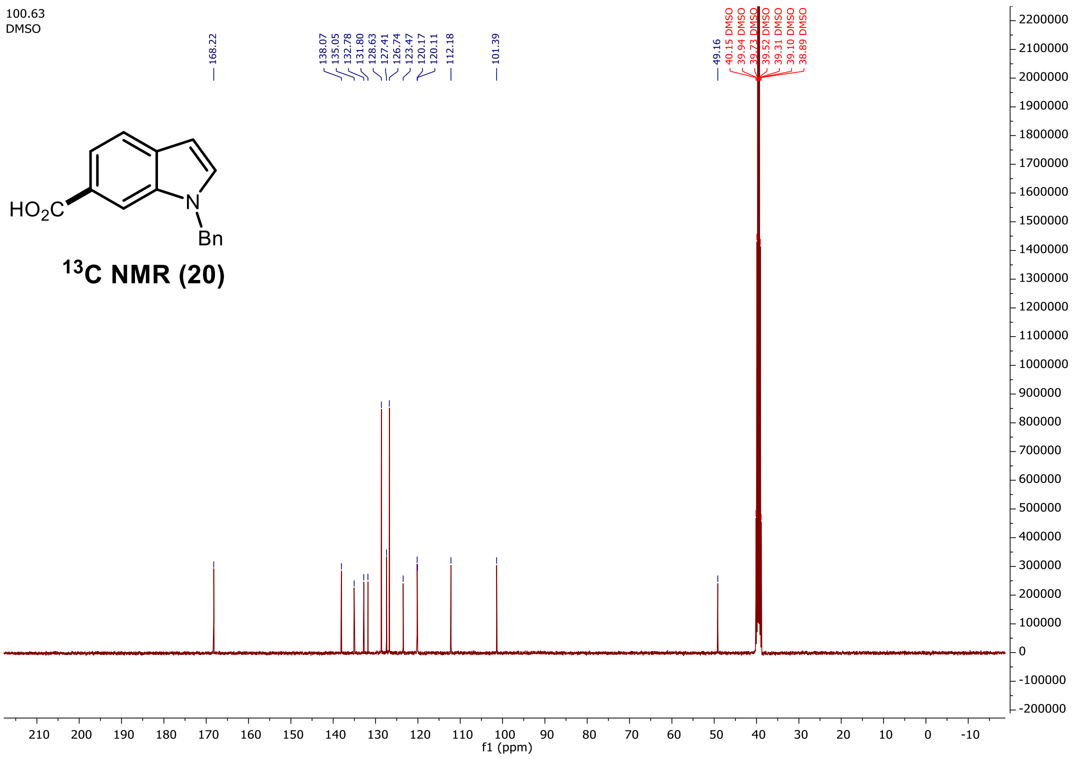
100.63
DMSO
168.22
138.07 135.05 132.78 131.80 128.63 127.41 126.74 123.47 120.17 120.11
112.18
101.39
49.16
40.15 DMSO 39.94 DMSO 39.73 DMSO 39.52 DMSO 39.31 DMSO 39.10 DMSO 38.89 DMSO
HO2C
N
Bn
13C NMR (20)
f1 (ppm)

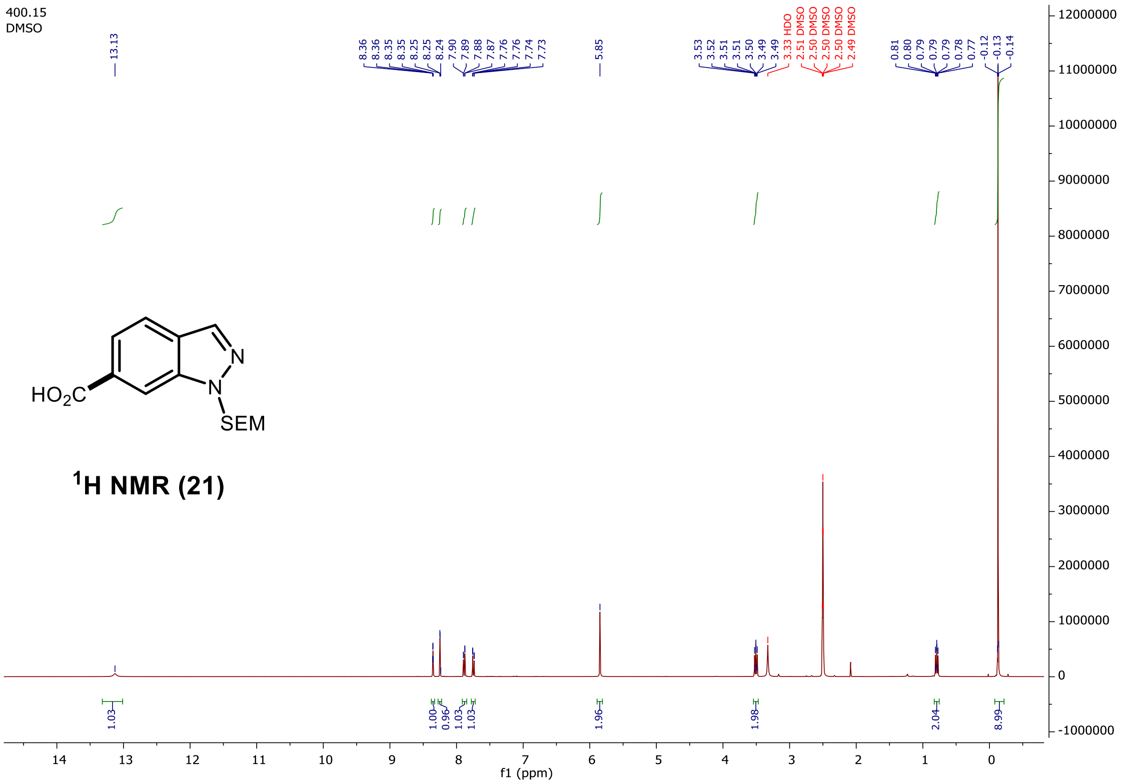
400.15
DMSO
HO2C
N
N
SEM
1H NMR (21)
f1 (ppm)

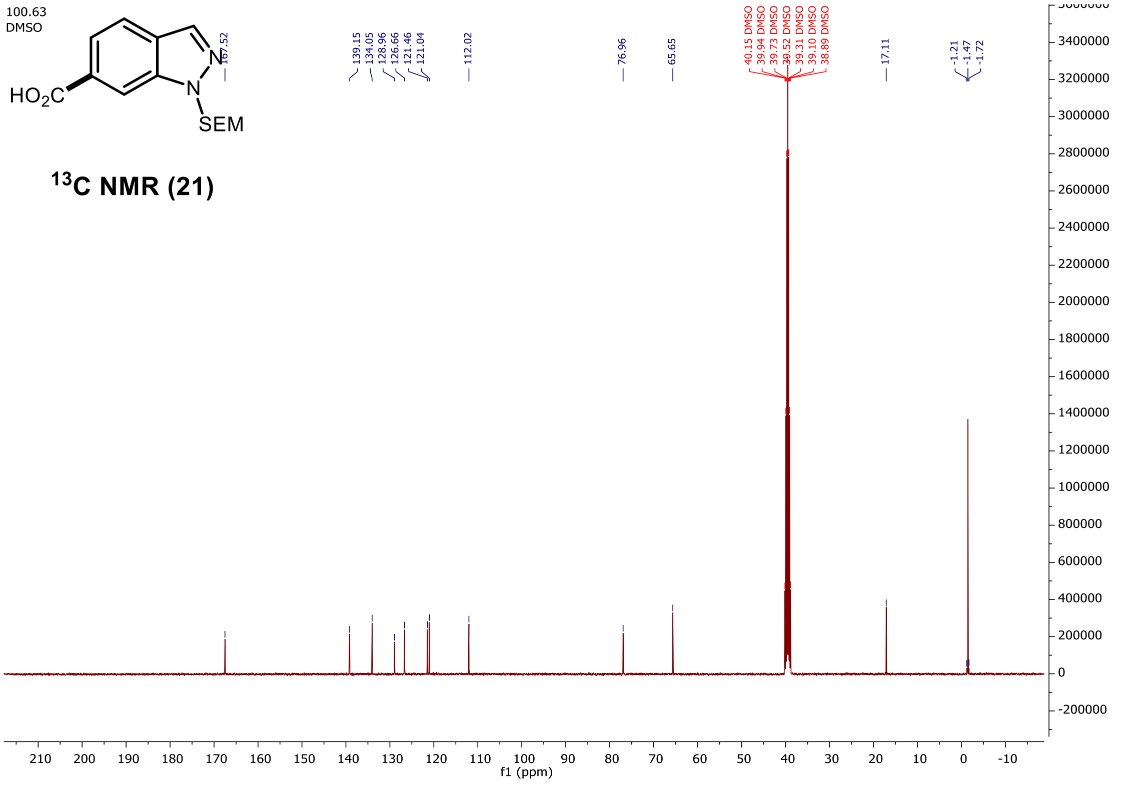
100.63
DMSO
HO2C
N
N
SEM
13C NMR (21)
f1 (ppm)
